# Red-light-activated and thermally-stable amido-dithienylethenes for the reversible control of G-quadruplex binding and anticancer activity

**DOI:** 10.1016/j.bioorg.2026.109710

**Published:** 2026-03-02

**Authors:** Nina M. Allen, Sam Bezzant, A. Sofia F. Oliveira, Javier Ramos-Soriano, M. Carmen Galan

**Affiliations:** a School of Chemistry, University of Bristol, Bristol BS8 1TS, United Kingdom; b Centre for Computational Chemistry, University of Bristol, Cantock’s Close, Bristol BS8 1TS, United Kingdom; c Instituto de Investigaciones Químicas (IIQ), CSIC – Universidad de Sevilla, Av. Américo Vespucio 49, Seville 41092, Spain

**Keywords:** G-quadruplex, G-quadruplex ligand, DTE, Photo-switch, Anticancer

## Abstract

Photo-responsive G-quadruplex (G4) ligands offer a powerful means to achieve spatiotemporal control over nucleic acid targeting, yet many existing scaffolds suffer from limited thermal stability or modest differences in affinity between photo-isomers. Here we report a thermally stable amido-pyridinium dithienylethene ligand (**2**) designed to enhance photo-isomer-dependent G4 recognition and increased structural modulation upon switching. Ligand **2** undergoes efficient and reversible photo-isomerization between its open and closed forms under near-UV and red-light irradiation, with long-lived photo-stationary states. Biophysical assays demonstrate that the open-isomer binds cancer-relevant G4 oligonucleotide sequences with 2–7-fold higher affinity than the closed form, while retaining high selectivity over duplex DNA. NMR studies reveal widespread perturbations across multiple G4 topologies, consistent with groove-associated interactions, and confirm that structural changes can be reversibly modulated by alternating irradiation. Importantly, the open-isomer exhibits a five-fold increase in cytotoxicity toward HeLa cells compared to the closed form, while showing negligible toxicity in healthy fibroblasts. Overall, ligand **2** represents a red-light-activated and thermally robust photo-switchable scaffold capable of reversible control over G4 binding and anticancer activity. These findings highlight the potential of amide-modified DTE frameworks as next-generation photo-responsive agents for precise regulation of G4-binding activity and anticancer activity.

## 1 Introduction

G-quadruplexes (G4s) are non-canonical nucleic acid structures forming in guanine rich sequences, which are key targets in the development of next-generation therapeutics. [1] G4-binding ligands have been reported as promising lead candidates for the treatment of a variety of human cancers, with demonstrated roles in the regulation of oncogene expression and telomerase activity. [2–4] Beyond these classical effects, G4 ligands have also been shown to engage additional cancer-relevant processes. For instance, some ligands inhibit ribosome biogenesis by stabilizing G4 structures in ribosomal DNA promoters, disrupting RNA polymerase I transcription and nucleolar function. [5] Certain compounds, such as pyridostatin, can induce DNA damage and genomic instability through stabilization of G4s and associated R-loops, resulting in double-strand breaks, checkpoint activation, and micronuclei formation. [6] Moreover, G4 stabilization can trigger innate immune responses by generating cytosolic DNA fragments that activate the cGAS-STING pathway, promoting anti-tumor immunity. [7] Most G4-binding chemotypes feature heteroaromatic rigid scaffolds decorated with cationic side chains designed to interact with G-quartets in a typical G4 sequence. [8] One challenge in the development of G4-ligands is the abundance of G4s across the human genome and transcriptome, making off-target activity and G4 topology discrimination a key concern. [4]

**Fig. 1:**
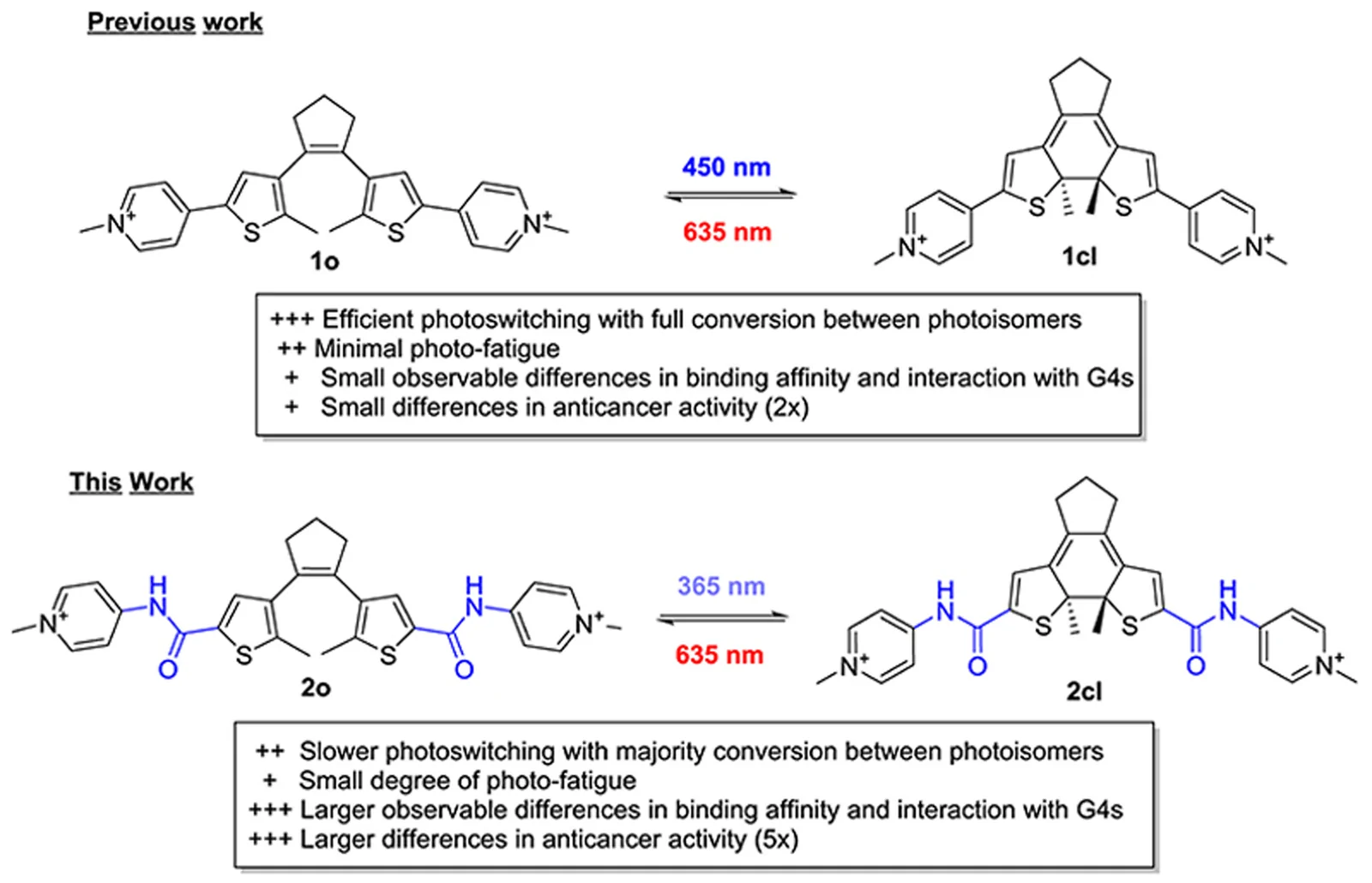
Dithienylethene ligands reported in the (a) previous [16] and (b) present studies.

To circumvent some of these issues, strategies that use an inactive form of the ligand that can be activated *in situ* to its active form, allows for precise spatiotemporal control of the ligand’s activity. Such approaches are commonly used in photo-pharmacology, whereby the non-invasive nature and low toxicity of light irradiation are particularly attractive. [9] In addition to classical irreversible photo-activation as used in photo-caging approaches, fully reversible photo-switchable ligands offer unique opportunities for increased control of activity, giving an on- and off-regulation. [9] Photo-switchable G4-ligands have been successfully employed for optically controlled G4-binding, anticancer activity and transcriptional control. [10–12] Previously reported photo-switchable G4-ligands have typically used stilbene- or azobenzene-based scaffolds which have large structural differences between the photo-isomeric forms, but are usually T-type photo-switches with low stability photo-isomers. [10] A key alternative scaffold is dithienylethenes (DTEs), which are generally P-type photo-switches with thermally stable photo-isomers. [13,14] Previous work from our group demonstrated the potential of the DTE scaffold to serve as the basis for photo-responsive and selective G4-binding ligands [15–17] and showed that the ligands’ cytotoxicity against cancer cells varied depending on the photo-isomeric state of the ligand. [16] However, whilst pyridinium functionalised DTE **1** (Fig. 1) displayed excellent photo-switchable activity and distinct binding modes with a model telomeric G4 sequence for the different photo-isomers, the isomers showed only minor differences in binding affinities, resulting in a two-fold difference in cytotoxicity between the photo-isomeric states.

We recently demonstrated in a series of azobenzene scaffolds that small structural changes in the ligand design, can have a big effect in binding affinity toward a specific G4 topology. [18] On this basis, we hypothesize that the introduction of an amide bond between the DTE core and the cationic motif will induce additional electronic and conformational changes in the ligand via intramolecular H-bonding, [19,20] and those differences should be further amplified in the open and close isomeric forms of the ligand, thereby affecting their binding to G4s.

Herein we report a novel amido-pyridinium DTE ligand **2** (Fig. 1), which exhibited demonstrable differences in G4-binding affinity toward cancer-related G4s between its open and closed isomeric forms. The ligand’s reversible light-induced structural effects on G4s were determined by NMR spectroscopy, and a 5-fold increase in cytotoxicity in HeLa cells for the red-light activated photo-stationary state was demonstrated.

## 2 Results and discussion

**Scheme 1:**
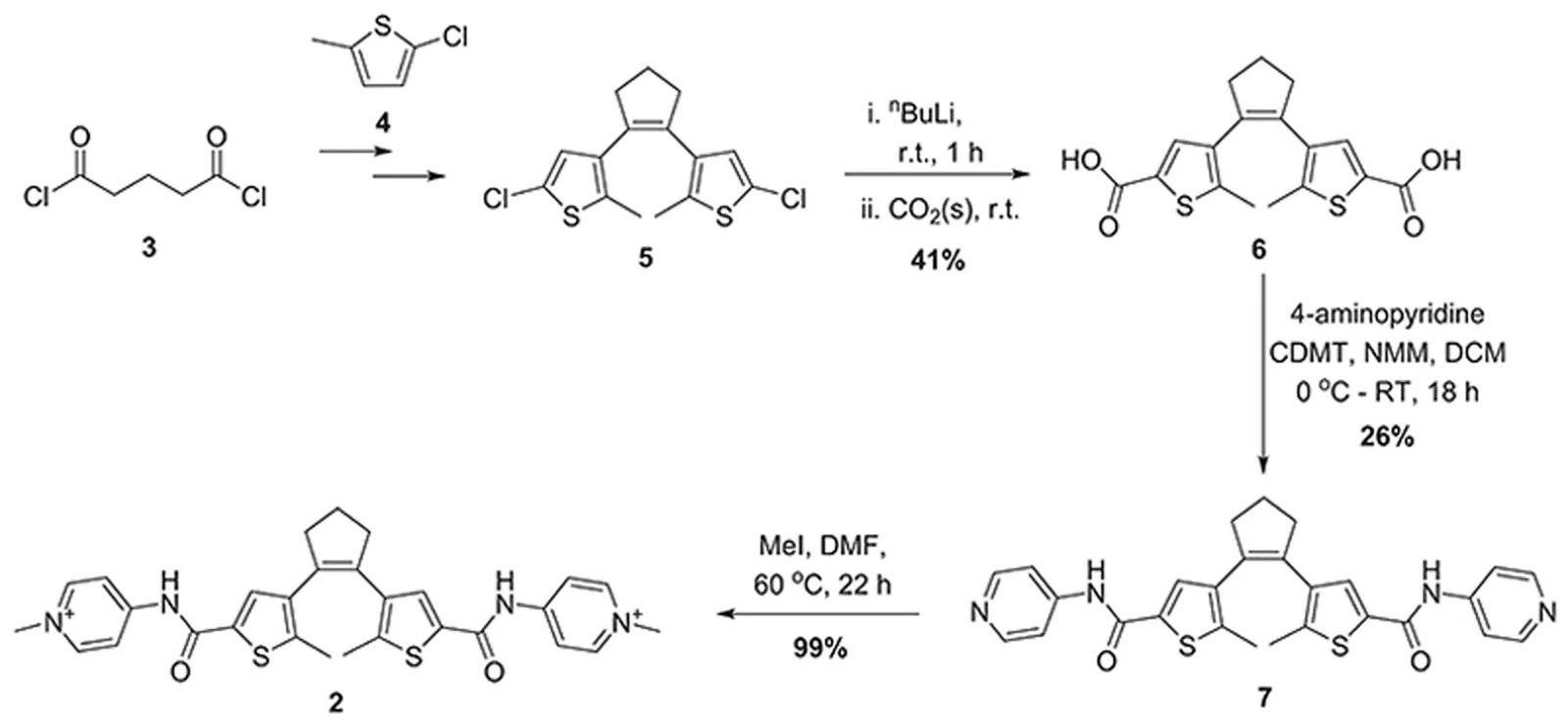
Synthesis of compound **2o**. NMM: *N*-methyl-morpholine; CDMT: 2-chloro-4,6-dimethoxy-1,3,5-triazine.

In order to obtain a novel DTE ligand with improved photophysical and biological properties compared to the parent DTE **1** (Fig. 1), we envisioned the synthesis of an amido-pyridinium ligand (**2**, Fig. 1) incorporating amide functionalities that could introduce structural diversification and potentially modify its properties via intramolecular H-bonding. The amido-pyridinium ligand **2o** was synthesized via a five-step procedure (Scheme 1). The positively charged pyridinium moiety was introduced to enable direct comparison with compound **1** and to evaluate the impact of amido modification on G4-binding and bioactivity. Briefly, dichloro-DTE **5** [16] was obtained by Friedel-Crafts acylation of glutaryl chloride (**3**) with 2-chloro-5-methylthiophene (**4**), followed by McMurray condensation. Carboxylic acid groups were introduced into compound **5** via lithiation with *^n^*BuLi, followed by reaction with solid CO_2_ to yield diacid **6**. [21] An amide-coupling step of **6** with 4-aminopyridine mediated by 2-chloro-4,6-dimethoxy-1,3,5-triazine (CDMT) and *N*-methyl-morpholine (NMM) then afforded compound **7** with a yield of 26%. The resulting amido-DTE **8** was then methylated in quantitative yield to give target compound **2o**.

**Fig. 2:**
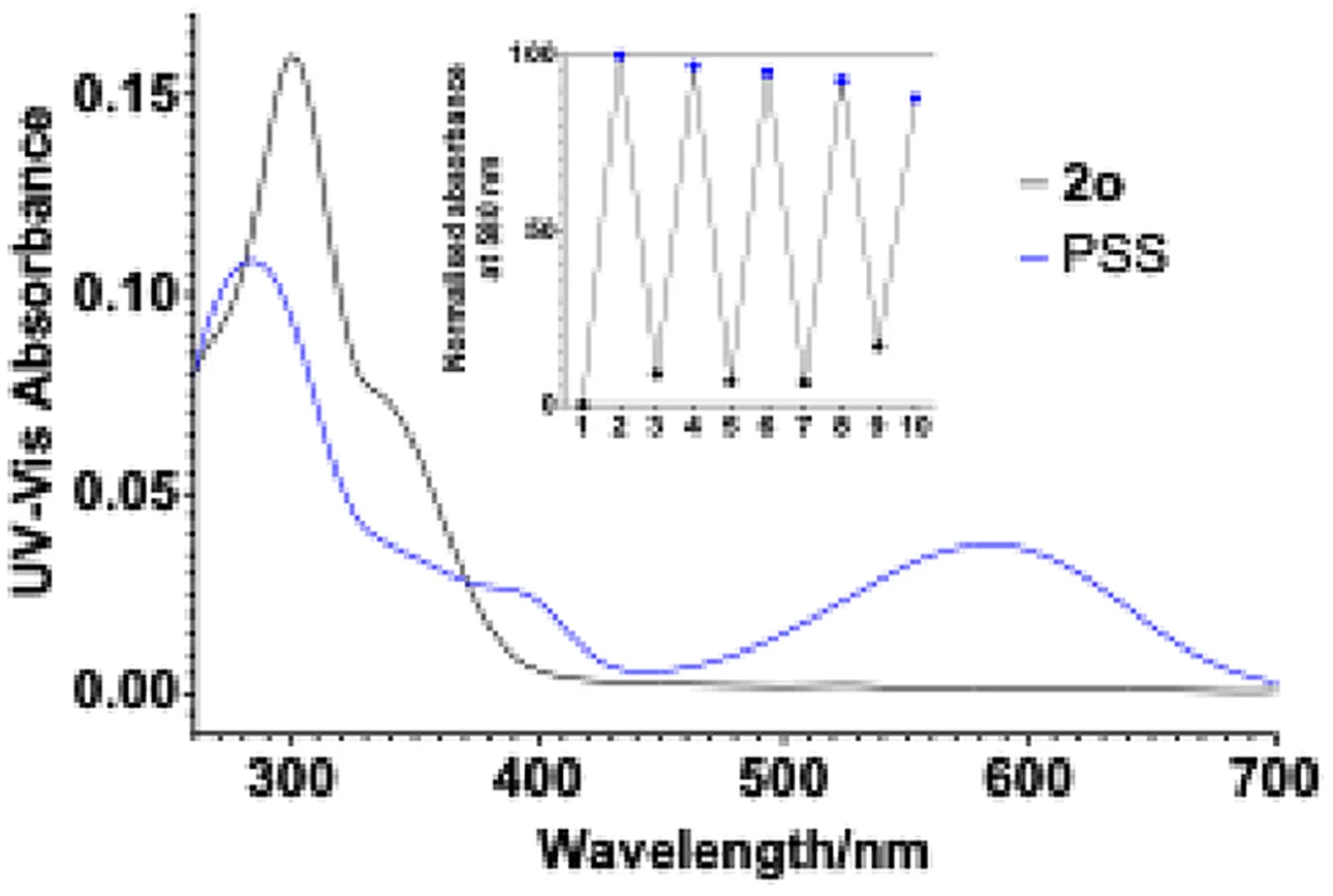
UV–Visible spectra of **2o** and photo-stationary state (1:5, **2o:2cl**) achieved under irradiation at 365 nm (100 μM in KPhos). The inset shows reversible switching over several cycles by alternate irradiation at 635 nm and 365 nm, monitored by absorbance at 580 nm.

With compound **2o** in hand, our initial investigations focused on evaluating its photophysical properties by UV–visible spectroscopy (Fig. 2). Upon irradiation with near-UV (365 nm), a blue shift of the absorption from ~300 nm to ~290 nm was observed, accompanied by the appearance of a new band centered around 580 nm. Subsequent exposure to red light (635 nm) for 30 min resuted in full reversion of the photo-stationary state (PSS) to **2o**. Reversible photo-switching between **2o** and PSS was observed (up to 10 switches with a small degree of photo-fatigue) by alternately irradiating the system with near-UV and red light (Fig. 2). NMR analysis indicated that, under near-UV irradiation in KPhos media, the PSS corresponds to a 1:5 ratio of **2o:2cl** (Fig. S5). Notably, the PSS remained stable in the dark at room temperature (t_1/2_ > > 48 h, Fig. S6).

**Fig. 3:**
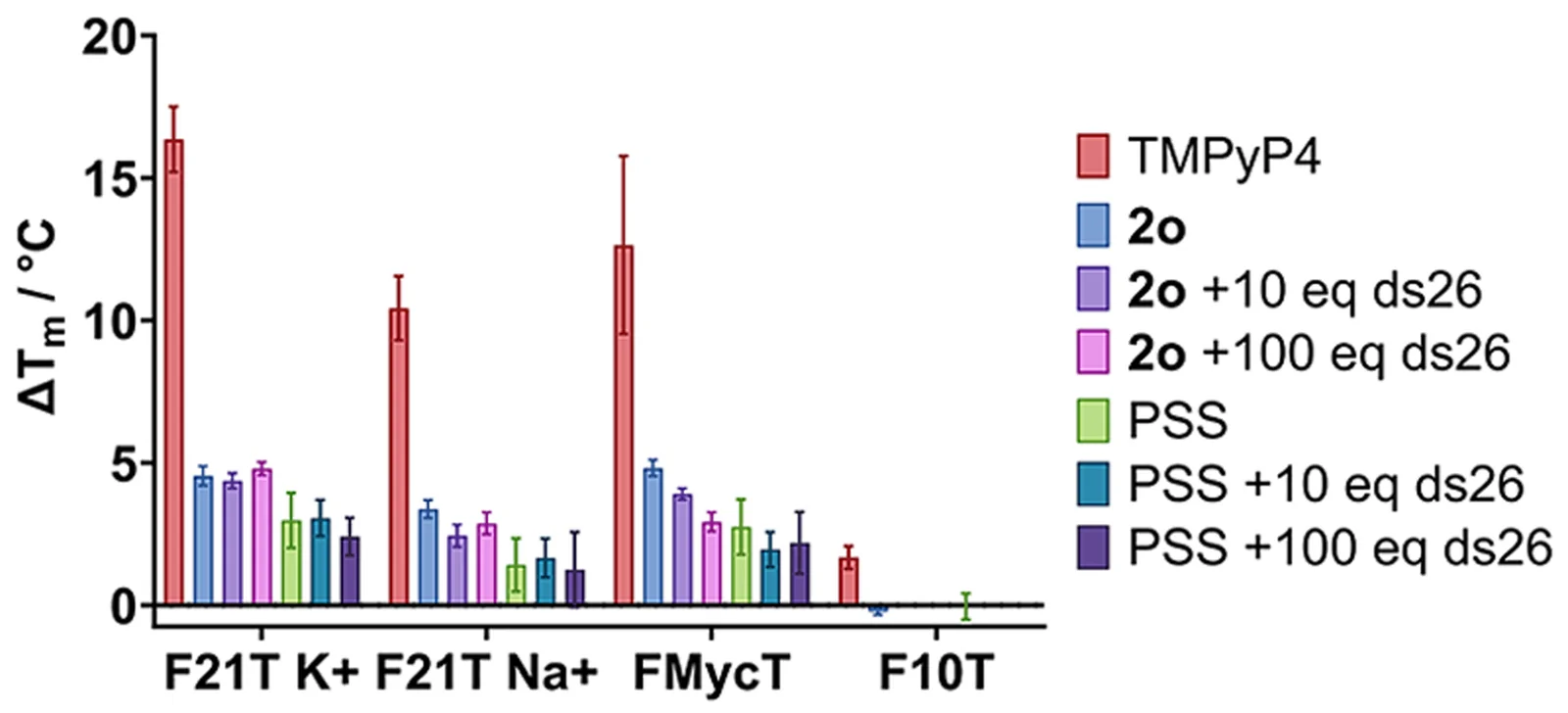
Induced thermal stabilization (ΔTm) by TMPyP4 (1 μM), **2o** (10 μM) and the PSS (10 μM) of a variety of cancer-derived G4s with increasing concentrations of a competing duplex sequence, monitored by FRET melting assay. Full data available in SI (Fig. S11–12).

To assess ligand selectivity toward G4s, three cancer-associated G4-forming sequences were chosen: an antiparallel 22-mer telomeric G4 annealed in a sodium-rich buffer (telo22), a predominantly hybrid 23-mer telomeric G4 (telo23), and a parallel 23-mer G4 from the c-Myc oncogene promoter region (c-Myc); and a duplex sequence (F10T) as control. The melting assay was carried out with both **2o** and the PSS, with increasing concentrations of a competing duplex sequence (ds26), to determine the selectivity for each of the G4s (Fig. 3). A small-degree of stabilization (up to 5 °C) was observed for G4-forming sequences tested, while no stabilization of the duplex sequence was observed. The stabilization for **2o** was slightly higher than for the PSS in all cases. Moreover, competition studies in the presence of up to 100 equiv. of duplex DNA did not show any changes in the ΔTm, suggesting a high degree of selectivity for the G4 sequences tested. It is worth noting that both **2o** and PSS showed evidence of thermal degradation at 95 °C, with degradation also occurring to a lesser extent at 60 °C and 40 °C (Fig. S7-S10), which could have a small effect the thermal stabilization observed.

To further investigate the interactions of the two photo-isomeric states with these G4 sequences, circular dichroism (CD), UV–visible, and NMR titrations were performed. CD titration experiments showed no significant topological changes for telo22, c-Myc and ds26 upon addition of either **2o** or the PSS (Fig. S13). In contrast, while the CD spectrum of telo23 remained unchanged in the presence of the PSS, the addition of ligand **2o** caused a decrease in ellipticity between 250 and 300 nm, suggesting conformational changes upon ligand binding (Fig. S13). Similar behaviour was previously observed for the non-amido DTE **1**, where the open-isomer **1o** exhibited comparable disruptive effects on telo23, [16] suggesting a potentially analogous binding mode for **2o**.

Despite the limited topological effects, UV–Visible titrations revealed notable differences in G4-binding affinity between the photo-isomeric states (Table 1 and Fig. S14). For the sequences tested, including ds26 duplex sequence for comparison, the binding affinity was 2–7 times higher for **2o** than for the PSS. Previously reported DTE-based G4 ligands did not exhibit such differences in affinity between isomers (see data in Table 1 for **1** and **2)**, [16] thus these results represent an important contribution toward the development of DTE-based photo-responsive G4 ligands. We hypothesize that the introduction of amide linkages can contribute to enhanced binding to G4 sequences by promoting additional stabilizing interactions with the nucleic acid backbone (see Docking section).

**Fig. 4:**
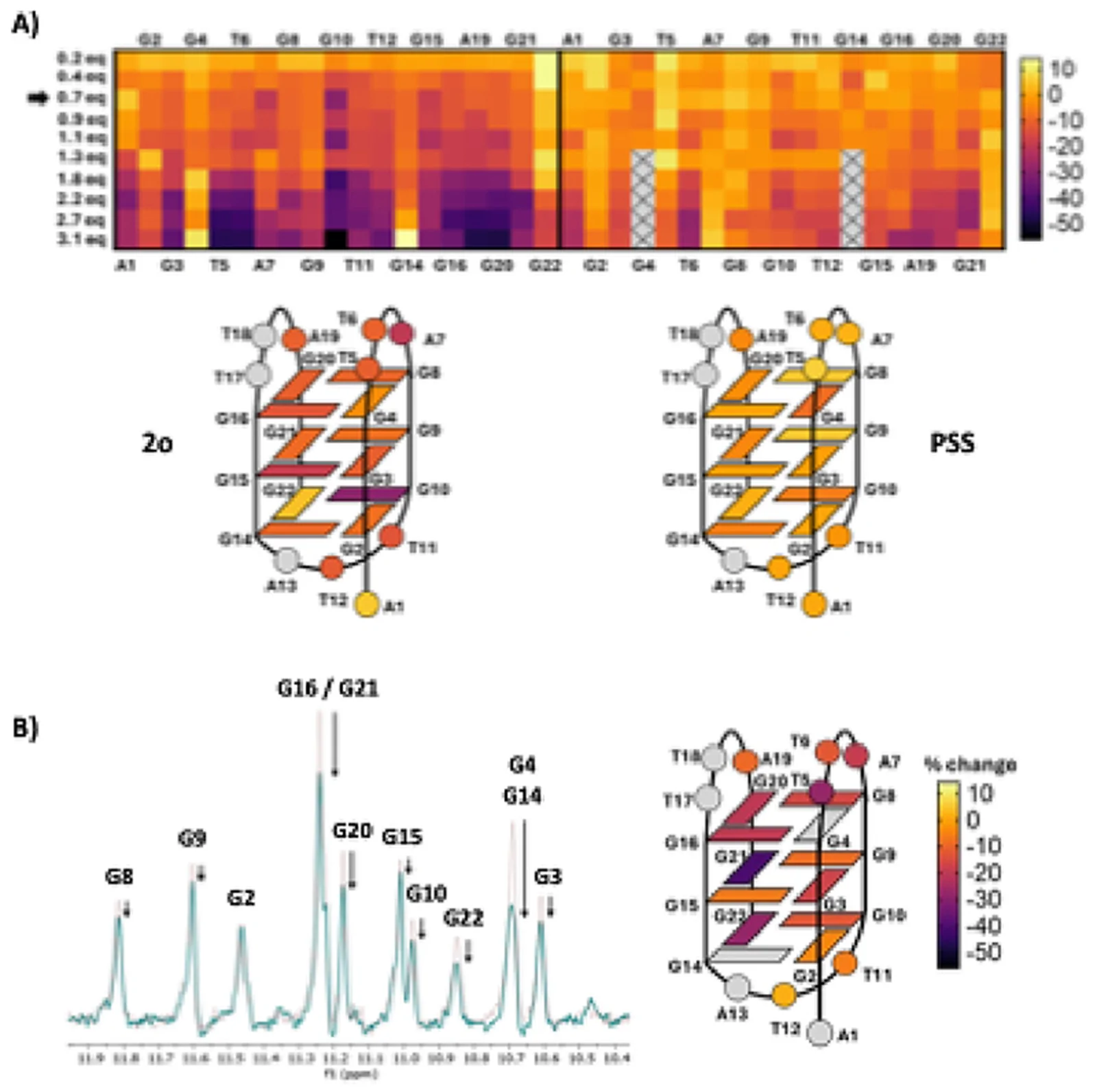
Heatmaps for **2o** and PSS showing percentage changes in the intensity of NMR peaks for protons associated with each base in the G4 structures. Below each heat map are representative structures showing the spatial orientation of the bases in each structure, overlayed with the percentage change in the intensity of the associated NMR peaks on addition of 0.7 eq of each ligand. Datapoints marked with an X indicate overlapping or otherwise obscured peaks where the intensity was not determined. (a) Data for titration of **2o** and the PSS into telo22 in NaPhos. (b) NMR spectra for telo22 in NaPhos, incubated with 3 eq of the PSS, pre- (red) and post-irradiation with 635 nm light. Annotated telo22 structure overlayed with colours representing the % change in the intensity of each NH resonance. Full assignments in Fig. S15. Full data in Fig. S17. (For interpretation of the references to colour in this figure legend, the reader is referred to the web version of this article.)

In order to gain more detailed insight into the binding mode, structural investigations via NMR spectroscopy were performed. The well-characterized antiparallel G4 telo22 in Na^+^ buffer [22] was first investigated since the association constants calculated from UV–Visible spectroscopy indicated a significant difference in the affinity of the two ligand forms. The acquired NMR data were consistent with these findings, with **2o** causing greater perturbations to the chemical shifts of the NH-resonances across the entire G4 structure (Fig. 4a). Both **2o** and the PSS induced shifts at the same resonances, albeit to different extents, suggesting that they do not possess unique binding modes. Since the PSS exists as a mixture of both photo-isomers, the spectral changes observed may arise from residual **2o** present in the mixture, while the closed-form may exhibit minimal observable interactions. Significant perturbations were detected for most assigned resonances, including those within top tetrad (G4, G8, G16 and G20), the central tetrad (G3, G9, G15 and G21) and the bottom tetrad (G2, G3 and G14). Additionally, perturbations were observed in both the top loops (T5, T6, A7, A19) and the bottom loop (T11 and T12). These data therefore suggest isomer **2o** may bind within one of the grooves, thereby causing widespread perturbations across the structure rather than π-stacking at the terminal tetrads. This interpretation aligns with the lack of topological changes observed via CD spectroscopy, as groove binders typically induce smaller CD variations than π-stacking or intercalative binders. [23] However, it should also be noted that although no induced CD effects-commonly associated with groove binders-were observed, [23,24] this does not preclude this binding mode as previously reported for other G4-targeting ligands. [25,26]

External control of the G4 structures upon interconversion between the photo-isomeric states was also investigated. Light-induced perturbations were observed (Fig. 4b), consistent with changes in the proportion of the more active **2o** isomer. Reversion of the PSS to **2o** upon irradiation with red light resulted in spectral changes consistent with the increased affinity of the open-isomer, with reduction in the intensity of several peaks. The most significant alterations occurred across the back face of the tetrad (particularly G20, G21 and G22), potentially indicating either a differential binding mode between the photo-isomers in this region or structural perturbations arising from the change in ligand geometry. Equivalent but opposite spectral changes were observed upon conversion of **2o** to the PSS using near-UV light (Fig. S17).

**Fig. 5:**
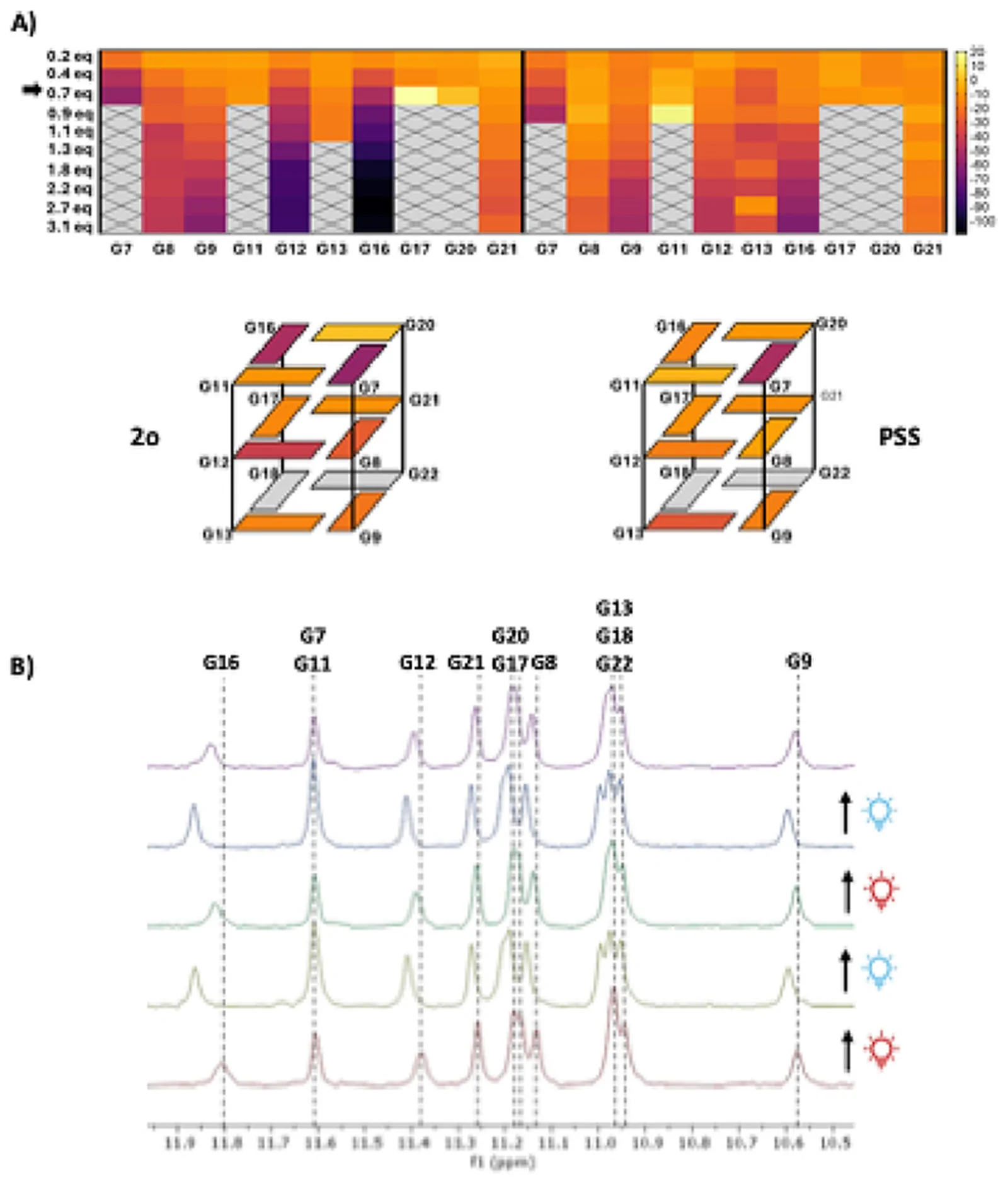
(a) Heatmaps for **2o** and PSS showing percentage changes in the intensity of NMR peaks for protons associated with each base in the G4 structures. Below each heat map are representative structures showing the spatial orientation of the bases in each structure, overlayed with the percentage change in the intensity of the associated NMR peaks on addition of 0.7 eq of each ligand. Datapoints marked with an X indicate overlapping or otherwise obscured peaks where the intensity was not determined. Data for titration of **2o** and the PSS into c-Myc in KPhos. (b) NMR spectra for c-Myc in KPhos, incubated with 3 eq of the PSS, with alternate irradiation with 635 and 365 nm light. Full assignments in Fig. S16. Full data in Fig. S18 in SI.

An analogous assay was performed for c-Myc, [27] for which the ligand exhibited the highest binding affinity (Fig. 5a). As observed for telo22, perturbations were detected throughout the structure for both **2o** and the PSS, with **2o** producing the largest spectral changes. The most pronounced interactions for **2o** were observed at G7 and G16 within the top tetrad, suggesting a possible π-stacking interaction at that face. The telo23 structure in KPhos was also examined with both ligands; however, a full resonance assignment was not possible due to the coexistence of multiple topologies (Fig. S19). Consistent with the binding affinities discussed above, titration of **2o** induced markedly greater spectral changes than the PSS. As c-Myc exhibited the most significant spectral changes of the sequences studied, a reversibility assay was conducted in which spectrum was recorded after alternating irradiation with 365 and 635 nm light (Fig. 5b). The spectrum displayed fully reversible changes, consistent with interconversion between the two photo-isomeric states inducing distinct degrees of structural perturbation.

**Fig. 6:**
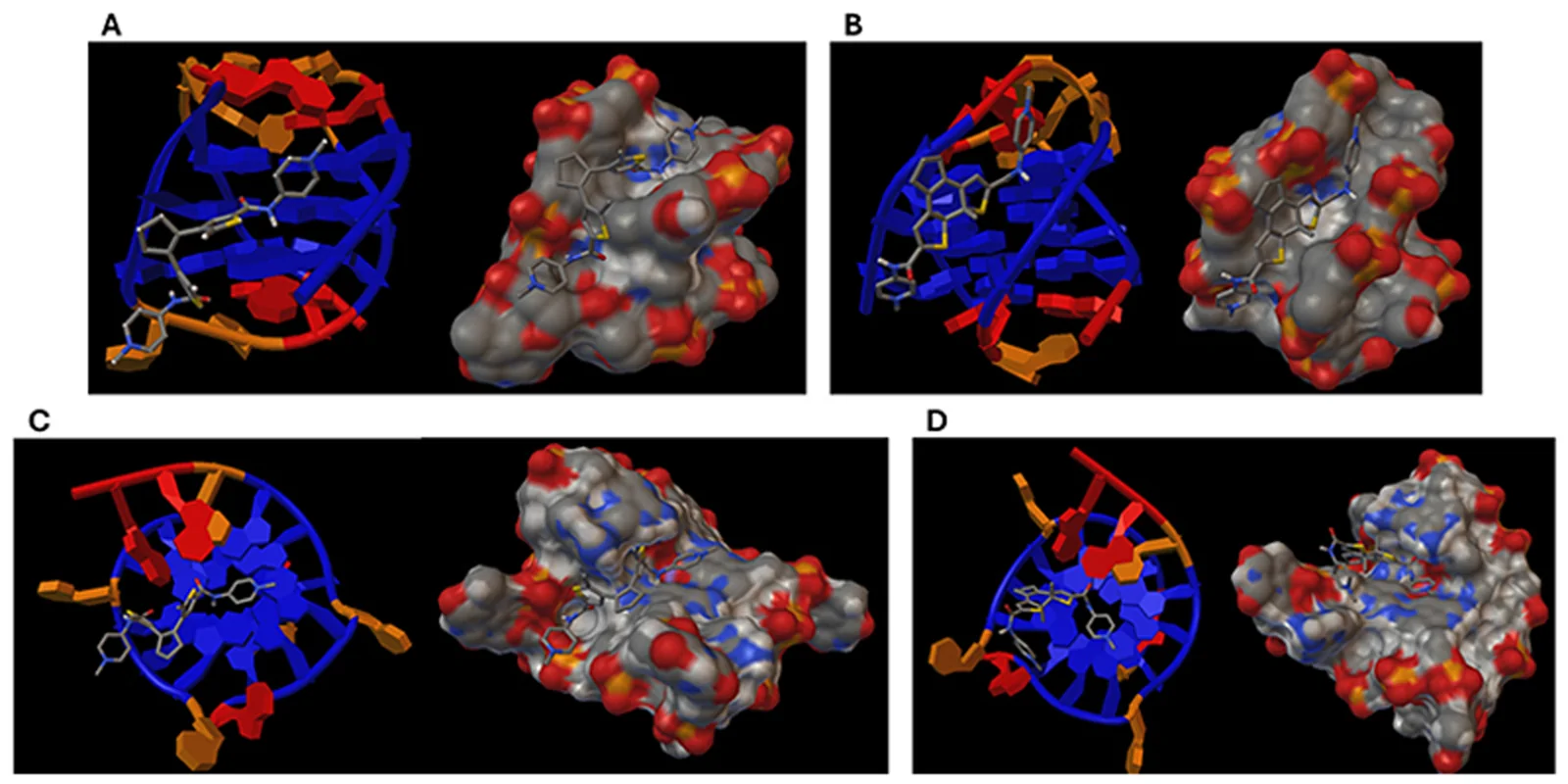
Examples of some of the most energetically favourable binding modes obtained from the docking calculations for telo22 (PDB: 143D) with **2o** (**A**) and **2 cl** (**B**) and c-Myc (PDB: 2LV7) with **2o** (**C**) and **2cl** (**D**). Each model is represented by a cartoon of the sequence coloured by base (red = adenine, orange = thymine and blue = guanine) and a molecular surface coloured by atom (grey = carbon/hydrogen, blue = nitrogen, red = oxygen and orange = sulfur). (Full modelling data is available in the ESI Fig. S21–24). (For interpretation of the references to colour in this figure legend, the reader is referred to the web version of this article.)

Molecular docking simulations were performed using AutoDock Vina [28] to identify the potential binding modes for the two photo-isomers when interacting with the antiparallel telo22 and parallel c-Myc G4 topologies, respectively (Fig. 6). In all cases, the relative energies of the eight lowest-energy binding poses were similar, suggesting the absence of a single, well defined high-affinity binding site in these systems. This observation aligns with the NMR titrations which showed widespread, minor perturbations, as well as limited changes in the CD spectra upon ligand titration. [29]

Docking studies with telo22 indicate that both photo-isomers primarily engage in groove-binding interactions, but with distinct binding preferences and interaction profiles (a and b, Fig. S21–S22 in SI). The open isomer **2o** adopts multiple stable binding modes, preferentially occupying the rear or right-hand grooves, where its bent and flexible structure enables interactions with both groove and loop nucleotides, such as particularly with A7 and A13 in the loops, G8–10, G20–22 on the rear face and G3-4 and G8-9 on the right face. These interactions include mainly end-face and offset π-stacking, along with extensive contacts with the sugar-phosphate backbone. In contrast, the closed isomer **2 cl** favors binding within the right or left groove (A1, T11 and T12 in the loops, G14-16, G20-21 in the left groove, and G2-G4 and G8-G10 in the right groove), displaying more peripheral interactions with fewer loop contacts and weaker π-stacking contributions, reflecting its flatter and more rigid structure.

For the c-Myc G4, [27,30] both photo-isomers exhibited multiple binding modes on the top and bottom faces (Fig. 6–c-d and Fig. S22–23 in SI). The open isomer **2o** showed similar stable binding to either face, with additional contacts to loop and groove regions facilitated by its greater flexibility. Generally, the ligands’ interactions were dominated by end-face and offset π-stacking, along with sugar–phosphate backbone contacts, rather than face-to-face π-stacking. In contrast, the more rigid closed **2cl** isomer also bound the top and bottom faces but showed fewer groove interactions and only limited face-to-face π-stacking.

Overall, the greater flexibility of the open isomer compared to the closed one allows for tighter and more extensive interactions with the both G4 structures, consistent with its higher binding affinity and greater structural perturbations observed experimentally. The docking results further suggest that the extended amide linkage in **2** enhances groove binding to telo22, with additional stabilization arising from amide-mediated hydrogen bonding to the sugar–phosphate backbone.

**Fig. 7:**
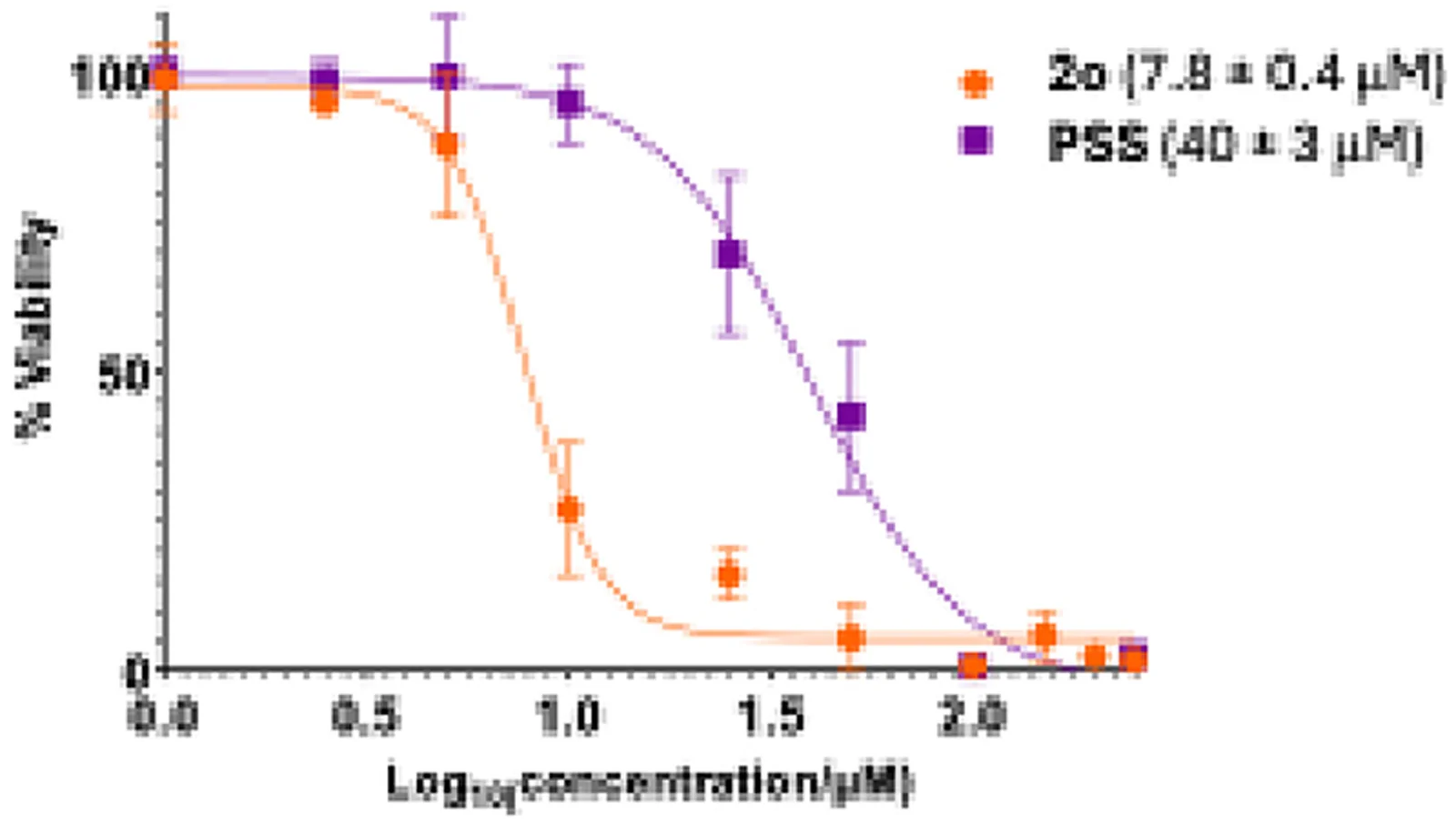
Viability assay data (IC50) for **2o** and **PSS** against HeLa cells after 72 h incubation. For comparison, IC_50_ values for HeLa cells treated with **1o** and **1c** under the same conditions were **1o** (23 ± 3 μM) and **1cl** (10 ± 1 μM).^[18]^ For both **2o** and the PSS the incubation was carried out in the dark, with each concentration achieved via dilution of pre-prepared samples of each photo-isomeric state (PSS was achieved by irradiation at 365 nm prior to dilution).

To evaluate the potential of this G4 ligand as an anticancer photo-responsive agent, cytotoxicity assays were performed in both healthy (HDF) and cancer (HeLa) human cell lines (Fig. 7) in the dark. Neither isomer showed appreciable cytotoxicity in the healthy cell line after 72 h (IC_50_ > 100 μM). In contrast, in HeLa cells, **2o** exhibited approximately five-fold greater toxicity (IC_50_ = 7.8 ± 0.4 μM) than the PSS (IC_50_ = 40 ± 3 μM) after 72 h (cf. IC_50_ **1o** = 23 μM, **1cl** = 10 μM) indicating that the toxic effect of the amido-DTE scaffold is dependent on the photo-isomeric form of the ligand, as we proposed. These results are consistent with the higher association constants of **2o** for G4s, suggesting that the observed cytotoxicity could potentially be G4-mediated. However, further studies would be needed to determine the mode of action of these ligands as their activity may also be attributable to other pathways.

## 3 Conclusions

In conclusion, we have demonstrated a thermally stable, photo-responsive DTE-based G4 ligand that exhibits red-light-triggered enhancement of G4 binding and anticancer activity. The open-form of the DTE displayed increased thermal stabilization, binding affinity, and structural perturbations with model cancer-related G4s compared with the PSS. The structural effects on the G4s were reversible over multiple irradiation cycles using near-UV and red-light.

These findings provide compelling evidence for the potential of amido-DTEs as a thermally stable, red-light-activated alternatives to azobenzene-based G4-photo-switches. This is particularly notable in the 5-fold increase in HeLa cell cytotoxicity observed for the open-isomer, while neither isomer exhibited toxicity in healthy HDF cells. Overall, these results demonstrate that amide incorporation into DTE-based scaffolds provides an effective strategy for fine-tuning photo-isomer-dependent G4 binding and enables precise, reversible optical regulation of anticancer activity. This amido-DTE platform thus represents a promising step toward the design of next-generation photo-responsive agents targeting G-quadruplex structures. Further studies in this area could enable the development of potent, photo-responsive G4 ligands offering enhanced spatio-temporal control compared with conventional therapeutics.

## 4 Experimental section

### 4.1 Chemistry

#### 4.1.1 General experimental methods

Reagents and solvents were purchased as reagent grade and used without further purification. For column chromatography, silica gel 60 (230–400 mesh, 0.040–0.063 mm) was purchased from E. Merck. Thin Layer Chromatography (TLC) was performed on aluminium sheets coated with silica gel 60 F254 purchased from E. Merck, visualization by UV light. NMR spectra were recorded on a Bruker Cryo 600 MHz instrument. All the assignments were confirmed by one- and two-dimensional NMR experiments (COSY, HSQC and HMBC). IR spectra were recorded on a Perkin Elmer Spectrum One FT-IR spectrometer fitted with a universal ATR accessory. The intensity of each band is described as s (strong), m (medium), w (weak) or sh (sharp) and with the prefix v (very) and suffix br (broad) where appropriate. Mass spectrometry was carried out by the University of Bristol Mass Spectrometry Service on a micrOTOF II (ESI) spectrometer, with the HRMS mode incorporating a lock-in mass injected midway through the run (sodium formate). Preparative HPLC was performed on a Grace Discovery Sciences Reveleris Prep System with a Phenomenex Luna 5 μm C18(2) 100 Å AXIA packed (250 × 21.2 mm) column. For purification, the instrument was set to monitor at 220 nm, 260 nm and 280 nm on the UV detector. Flow rates were set at 14 mL/min.

#### 4.1.2 Synthesis and characterization

*4,4′-(cyclopent-1-ene-1,2-diyl)bis(5-methyl-N-(pyridin-4-yl)thiophene-2-carboxamide) (**7**)*: To a solution of 4,4′-(cyclopent-1-ene-1,2-diyl)bis (5-methylthiophene-2-carboxylic acid) **6** (216 mg, 0.61 mmol) in anhydrous DCM (20 mL), cooled to 0 °C, *N*-methyl-morpholine (0.12 mL, 1.1 mmol) and 2-chloro-4,6-dimethoxy-1,3,5-triazine (200 mg, 1.1 mmol) were added. The reaction mixture was stirred at 0 °C for 90 min. *N*-methyl-morpholine (0.12 mL, 1.1 mmol) and 4-aminopyridine (100 mg, 1.1 mmol) were then added and the mixture stirred at 0 °C for 2 h then at r.t. for 16 h. The solution was then diluted with DCM, washed with aq. 1 M HCl (x2), brine, saturated aq. NaHCO_3_ and H_2_O. The aqueous layers were then back extracted with DCM (x2). The combined organic layers were dried over anh. MgSO_4_, filtered and concentrated under reduced pressure. The resultant residue was then purified by silica flash column chromatography (25–100% CHCl_3_ in hexane then 0–20% MeOH in CHCl_3_) to yield **7** (82 mg, 26%) as a brown solid. ^1^H NMR (500 MHz, CDCl_3_) *δ* 8.28 (d, *J* = 6.1, 4H, H-12), 7.67 (d, *J* = 6.2, 4H, H-11), 7.51 (s, 2H, H-2), 2.69 (t, *J* =7.5, 4H, H-7), 2.08–1.99 (m, 2H, H-8), 2.03 (s, 6H, H-5); ^13^C NMR (125 MHz, CDCl_3_) *δ* 160.9 (C-9), 149.1 (C-12), 146.7 (C-10), 142.7 (C-1/3/4), 136.8 (C-1/3/4),135.2 (C-6), 134.1 (C-1/3/4), 131.5 (C-2), 114.3 (C-11), 38.2 (C-7), 22.9 (C-8), 14.8 (C-5); HRMS (ESI^+^): [M + 2H]^2+^ calculated for C_27_H_26_N_4_O_2_$S_{2}^{2+}$: 251.0742, observed 251.0741; IR ν_max_ (cm^−1^) (thin film): 2921 (s), 2851 (m), 1669 (m), 1591 (s), 1509 (s), 1456 (m), 1416 (m), 1330 (m).

*4,4′-((4,4′-(cyclopent-1-ene-1,2-diyl)bis(5-methylthiophene-4,2-diyl-2-carbonyl))bis(azanediyl))bis(1-methylpyridin-1-ium) (**1**): **7*** (25.0 mg, 50 μmol) was dissolved in DMF (1 mL) and IMe (0.2 mL) and stirred 60 °C for 22 h and then concentrated under reduced pressure. The residue was suspended in 1:1 hexane:EtOAc and filtered, the solid was washed with 1:1 hexane:EtOAc yielding compound **1** (38.6 mg, 99%) as a brown solid. ^1^H NMR (600 MHz, DMSO-*d*_6_) *δ* 11.31 (s, 2H, NH), 8.74 (d, *J* =7.2, 4H, H-12), 8.22 (d, *J*= 7.0, 4H, H-11), 8.03 (s, 2H, H-2), 4.19(s, 6H, H-13), 2.88 (t, *J* = 7.4, 4H, H-7), 2.12 (p, *J* = 7.5, 2H, H-8), 1.93 (s, 6H, H-5); ^13^C NMR (151 MHz, DMSO-*d*_6_) *δ* 161.2 (C9), 152.1 (C-10), 146.3 (C-12), 144.1 (C-1/3/4), 137.2 (C-1/3/4), 134.9 (C-6), 134.1 (C-1/3/4), 132.9 (C-2), 115.8 (C-11), 46.9 (C-13), 39.0 (C-7), 23.0 (C-8), 15.1 (C-5); HRMS (MALDI): [M-H]^+^ calculated for C_29_H_29_N_4_O_2_$S_{2}^{2+}$: 529.1726, observed: 529.1721; IR ν_max_ (cm^−1^) (thin film): 3382–2439 (br m), 1716 (m), 1669 (s), 1640 (m), 1590 (m), 1519 (s), 1461 (s), 1323 (m).

### 4.2 Photophysical experiments

#### 4.2.1 Photo-switching investigations by UV–visible spectrometry

UV–Visible spectra were recorded using an Agilent Cary 60 UV–Vis Spectrophotometer at ambient temperature using a room-light immune fibre optic probe with 10 mm path length/or using a quartz glass cuvette with a 10 mm path length. UV–visible spectra were recorded between 800 and 200 nm and baseline corrected for the buffer used. UV–Visible spectroscopy was used to determine suitable wavelengths for the interconversion of the photo-isomers of **2** (100 μM, KPhos water). Irradiation at 365 nm (Analytikjena UVP 3UV Lamp, 10 cm distance from lamp) resulted in a shift at the peak ~300 nm to ~290 nm, and the appearance of a peak at ~580 nm. The PSS could be achieved after 30 min of irradiation at 365 nm – as no further changes of the spectrum were observed after further irradiation beyond this time point. Full reversion of the PSS to **2o** was observed after 30 min irradiation with 635 nm light. To assess photo-fatigue of **2**, UV–Visible spectra were recorded after alternate irradiation with 365 nm and 635 nm light (ThorLabs 635 nm 4.5 mW collimated laser diode module), with irradiation at each wavelength continued until no further spectral changes were observed. Monitoring at 580 nm allowed for determination of the proportion of each photo-isomeric state present after irradiation. While photo-switching was observed up to 10 cycles, a degree of photo-fatigue was observed, particularly for induction of the PSS, with a consistent decrease in the absorbance at 580 nm after each cycle.

#### 4.2.2 Determination of the nature of the PSS by NMR spectroscopy

NMR spectroscopy (Prodigy 600 MHz NMR spectrometer with a room temperature probe) of **2** (500 μM, KPhos +10% D_2_O) was used to determine the ratio of **2o:2 cl** in the PSS. The PSS was reached in ~1 h under 365 nm irradiation, and determined to consist of a 1:5 ratio of the **2o:2 cl**. Further irradiation did not result in further conversion of **2o** to **2 cl**, but no side products were observed.

#### 4.2.3 Thermal relaxation of the PSS by UV–visible spectrometry

To assess thermal relaxation of the PSS, UV–Visible spectra of the PSS (100 μM, HPLC water) were recorded in the dark at r.t. over 48 h. Monitoring at 580 nm allowed for determination of the remaining PSS present. No evidence of thermal relaxation was observed under these conditions.

#### 4.2.4 Thermal degradation at high temperatures

To assess thermal degradation of **2o** and the PSS, UV–Visible spectra of the **2o** and the PSS (100 μM, HPLC water) were recorded in the dark at 95 °C for up to 2 h. In both cases reduction in all compound peaks were observed combined with a new peak forming at 260 nm. An aliquot of the endpoint was then doped with D_2_O and the ^1^H NMR spectrum was recorded (Prodigy 600 MHz NMR spectrometer with a room temperature probe). In both cases the formation of a new species distinct from either isomer was observed, suggesting thermal degradation of both 2o and the PSS at 95 °C.

### 4.3 Biophysical experimental section

All oligonucleotides used were purchased from Eurogentec (Belgium), purified by HPLC and delivered dry. Oligonucleotide concentrations were determined by UV-absorbance using a EzDrop1000 spectrophotometer. Oligonucleotides were annealed by heating to 90 °C for 2 min and then cooled on ice for at least 10 min. Topology was determined by CD spectroscopy.

#### 4.3.1 FRET melting experiments

FRET melting assays were performed according to the procedure reported by De Cian et al. [31] on a AriaMx qPCR instrument. DNA sequences were obtained labelled with FAM (fluorescence donor) and TAMRA (fluorescence quencher) at the 5′ and 3′ ends. The final concentration in each sample was 200 nM. Fluorescence was recorded over a temperature range of 25–96 °C in 1 °C/min increments (after holding for 5 min at 25 °C). Each sample was tested in duplicate and the experiments repeated 3 times.

The thermal stabilization of both **2o** and the PSS were calculated with 0, 10 and 100 eq of a competing duplex sequence (ds26), for 4 DNA sequences chosen for further investigation.

The data was processed and analysed using Python and Excel and plotted in GraphPad Prism. The melting temperature defined and calculated as the maximum of the first differential of the fluorescence vs temperature data. The change in melting temperature was then calculated as the difference in melting temperature between samples with or without the presence of the ligand.

#### 4.3.2 Circular dichroism for binding studies

CD spectroscopy titrations were recorded using a Jasco J-810 spectrometer fitted with a Peltier temperature controller. Measurements were taken in a quartz cuvette with a path length of 5 mm, at 20 °C, at a 50 nm / min scanning speed at 1 nm intervals, with a 1 nm bandwidth for 3 accumulations. The CD spectrum was recorded between 200 and 600 nm, and baseline corrected for the buffer used. DNA was used at a concentration of 5 μM in KPhos (c-Myc, telo23, ds26) or NaPhos (telo22). Data processing was carried out in Python and GraphPad Prism with second order polynomial smoothing applied to all spectra.

#### 4.3.3 UV–visible spectra for binding studies

UV–Visible spectra were recorded using an Agilent Cary 60 UV–Vis Spectrophotometer at ambient temperature using a room-light immune fibre optic probe with 10 mm path length/or using a quartz glass cuvette with a 10 mm path length. UV–visible spectra were recorded between 800 and 200 nm and baseline corrected for the buffer used.

For titrations, the concentration of the ligand was fixed at 10 μM with aliquots of oligonucleotide added using a microsyringe from a 100 μM stock solution in appropriate buffer also containing 10 μM ligand to maintain constant ligand. The solution was briefly stirred after each addition. Data were fitted to an appropriate binding model using non-linear regression in GraphPad Prism to provide estimates of the association constants.

#### 4.3.4 NMR experiments for binding studies

^1^H NMR spectra were recorded at 298 K on a 600 MHz Bruker Varian spectrometer. The DNA concentration was ~170–180 μM in KPhos (c-Myc, telo23) or NaPhos (telo22) containing 10% D_2_O. All experiments employed sculpted excitation water suppression. During the titrations, aliquots of ligand were added from a 10 mM stock solution in DMSO-*d*_6_ to give the required titration points. After addition of ligand aliquots, the sample was mixed thoroughly and the NMR spectrum acquired immediately. The resonances were assigned by comparison with previously published data. All spectra were processed with MestReNova software, including calculation of the intensities of each separable peak. The percentage changes of the intensities of each resonance were plotted in heat maps using GraphPad prism to give graphical representations of the most significant structural perturbations.

### 4.4 Molecular docking simulations

The three-dimensional structures of the **2o** (open) and **2 cl** (closed) photo-isomers were first constructed using GaussView 6.1.1, [32] then subjected to an initial geometry optimization using molecular mechanics (with Gaussian 16 using the UFF forcefield [33]), followed by refinement using DFT calculations (Gaussian 16 [32], B3LYP functional, 6-31 g (d) basis set).

For each photo-isomer, two conformations were constructed, differing by whether the amide group adopted a cis or trans relative to the thiophene ring (see ESI Fig. S20 for full details). The geometry-optimised structures (ESI Fig. S20) were subsequently docked into an antiparallel telo22 (PDB: 143D) [22] and a parallel c-Myc (PDB: 2L7V) [34] G4 structures, using AutoDock Vina[28]26] employing standard settings, except for the exhaustiveness value that was set to 1000. AutoDockTools [35] was used to prepare the G4 structures and ligands and convert their PDB files into PDBQT format. The numbers of xyz grid points in the docking boxes were 60, 80 and 64 for telo22 and 84, 86 and 60 for c-Myc, respectively, with a grid spacing of 0.375 nm. Two docking runs were performed for each G4 (PDB IDs 1I43 and 2L7V), and the eight best-scoring (i.e. lowest-energy) binding modes were visualized using Python Molecule Viewer 1.5.7 [36] (Fig. S21-S24).

### 4.5 Biology experimental section

HeLa cells were maintained at 37 °C in an atmosphere of 5% CO_2_ in DMEM high glucose (4.5 g/L) media supplemented with 10% FBS and 1% antibiotic-antimycotic cocktail. HDF cells were maintained at 37 °C in an atmosphere of 5% CO_2_ in MEM media supplemented with 10% FBS and 1% antibiotic-antimycotic cocktail.

Cells plated in 96-well plates in a volume of 100 μL with 2 ×10^4^ HeLa cells/ HDF cells per well. Cytotoxicity was determined by assay Alamar Blue. After desired incubation time with compound in the dark, media was removed and a solution of Alamar blue (5%) in serum-free media was added to each well and incubated for 2–6 h at 37 °C in an atmosphere of 5% CO_2_. Fluorescence of the dye measured was on a BMG Labtech CLARIOstar plate reader. The non-linear dose-response fitting tool in Prism was used to estimate IC_50_ values.

## Supplementary Material

Supplementary data to this article can be found online at https://doi. org/10.1016/j.bioorg.2026.109710.

Appendix A. Supplementary data

## Figures and Tables

**Table 1:** Induced thermal stabilization (ΔTm) and binding constants (K_a_) calculated from UV–Vis titrations carried out with antiparallel G4-forming sequence telo22, hybrid G4-forming sequence telo23, parallel G4-forming sequence c-Myc and duplex sequence ds2 for DTE **1**[16] and **2**.

		DTE 1		DTE 2	
Parameter	Sequence	1o	1c	2o	PSS (2o:2 cl, 1:1)
	F10T	1 ± 0.1	1 ± 0.3	–0.2 ± 0.3	0.0 ± 0.5
ΔT_m_ at 10 μM / °C	F21T Na^+^	4 ± 0.5	4 ± 0.3	3 ± 1	1.7 ± 0.7
	F21T K^+^	10 ± 0.2	13 ± 0.5	5 ± 1	1.9 ± 6
	FMycT	–	–	5 ± 1	3 ± 1
	Telo22	0.17 ± 0.03	0.14 ± 0.03	0.36 ± 0.01	0.08 ± 0.03
	Telo23	0.17 ± 0.03	0.17 ± 0.03	0.90 ± 0.05	0.36 ± 0.03
K_a_ / μM^–1^	c-Myc	–	–	1.7 ± 0.1	0.74 ± 0.04
	ds26	–	–	0.43 ± 0.08	0.06 ± 0.03

Note: Analysis for **2** was carried out at the wavelength of maximal change over the course of the titration (340 nm for **2o** and 580 nm for the PSS).

## Data Availability

Data will be made available on request.
