# Supplementary material for: Red-light-activated and thermally-stable amido-dithienylethenes for the reversible control of G-quadruplex binding and anticancer activity
Source: Bioorg Chem. Author manuscript; Available in PMC 2026 Oct 9. (PMC7619534; doi:10.1016/j.bioorg.2026.109710)
Supplement: Supplementary file 1 [file EMS218229-supplement-Appendix_A__Supplementary_data.pdf]

Red-Light-Activated and Thermally-stable Amido-Dithienylethenes for the Reversible Control of G-Quadruplex Binding and Anticancer Activity

Nina M. Allen[1], Sam Bezzant[1], A. Sofia F. Oliveira,[1,2] Javier Ramos-Soriano[3]* and M. Carmen Galan[1]*

[1] *School of Chemistry, University of Bristol, Bristol BS8 1TS, United Kingdom.*

[2]*Centre for Computational Chemistry, University of Bristol, Cantock's Close, Bristol BS8 1TS, United Kingdom*

[3]*Instituto de Investigaciones Químicas (IIQ), CSIC – Universidad de Sevilla, Av. Américo Vespucio 49, Seville 41092, Spain*

*corresponding authors: fj.ramos@iiq.csic.es and m.c.galan@bristol.ac.uk

Contents

Compound characterization 4
Thermal relaxation of the PSS at room temperature: 5
Thermal degradation **2o** at 95 °C: 5
Thermal degradation **PSS** at 95 °C: 6
Nucleic Acid Binding Studies 7
Materials used 7
CD spectroscopy titrations. 9
UV-Visible spectroscopy titrations. 10
NMR spectroscopy titrations. 11
Molecular Docking Calculations 14
References. 20

Compound Characterisation.

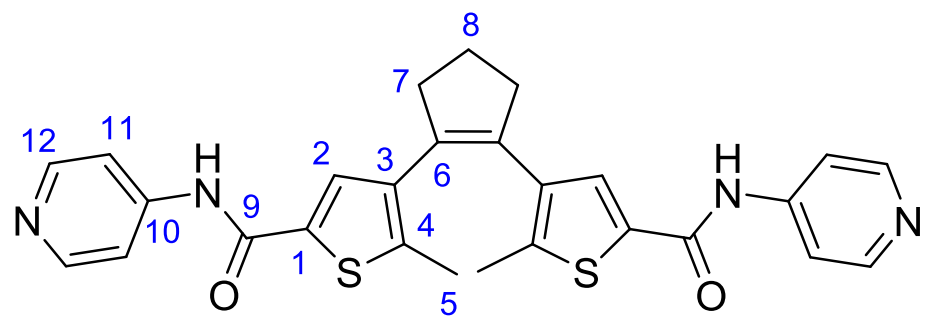

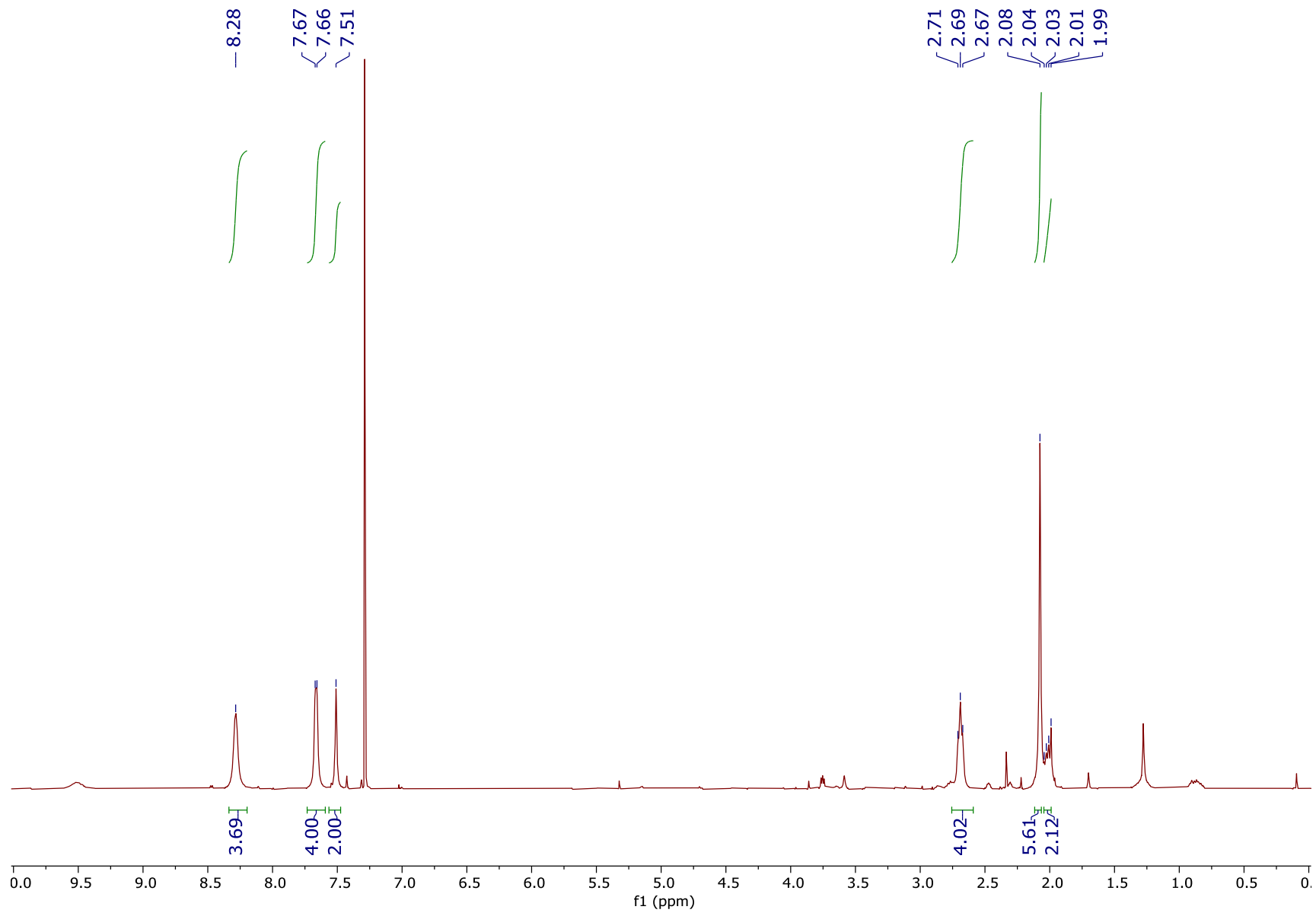

Figure S1. ^{1}H NMR spectrum of **7**

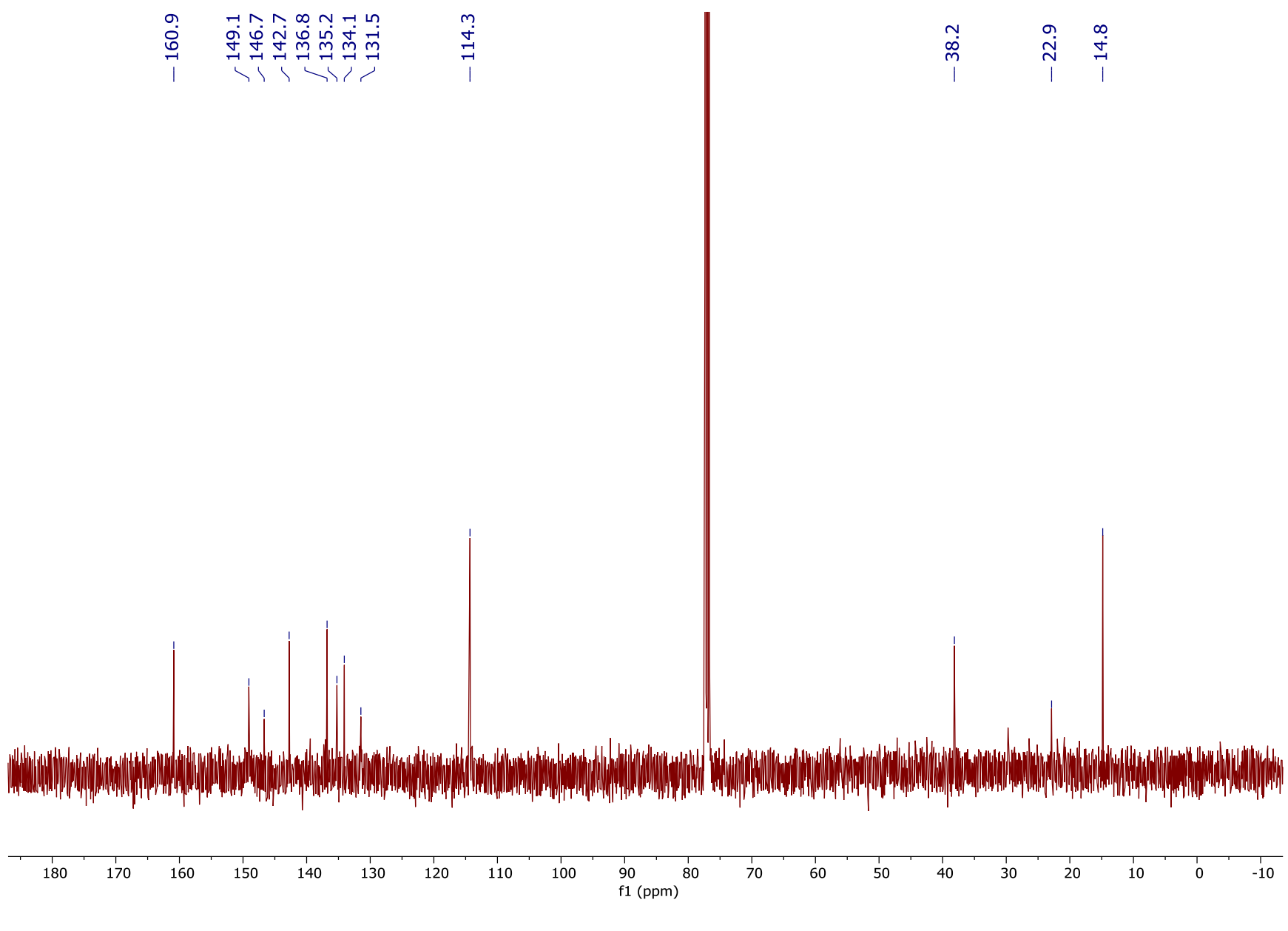

Figure S2. ^{13}C NMR spectrum of **7**

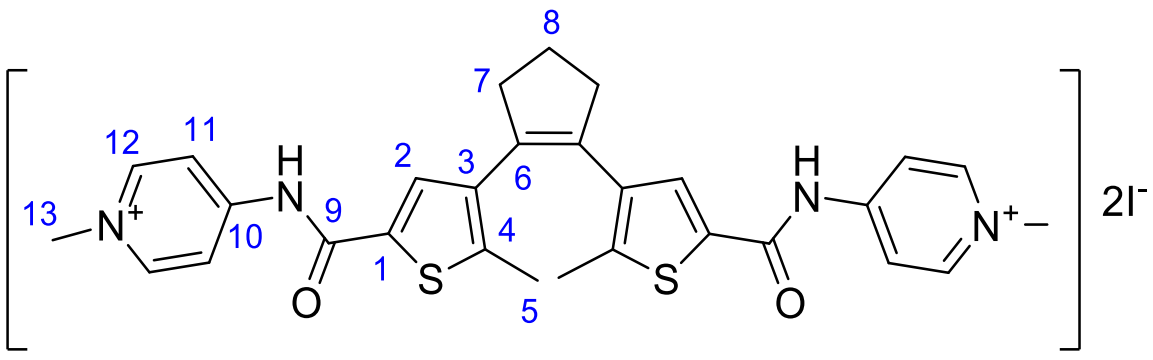

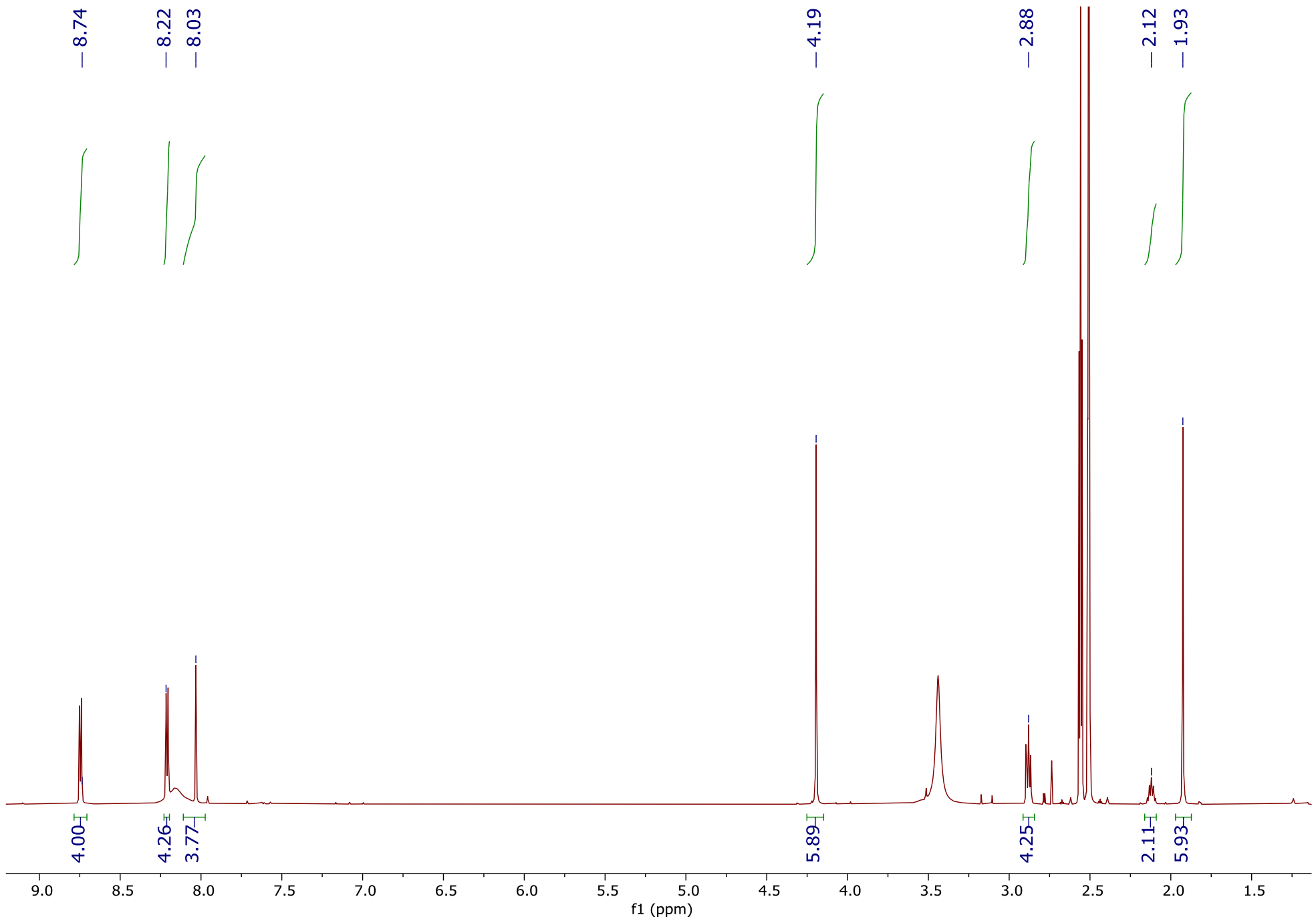

Figure S3. ^{1}H NMR spectrum of **2o**

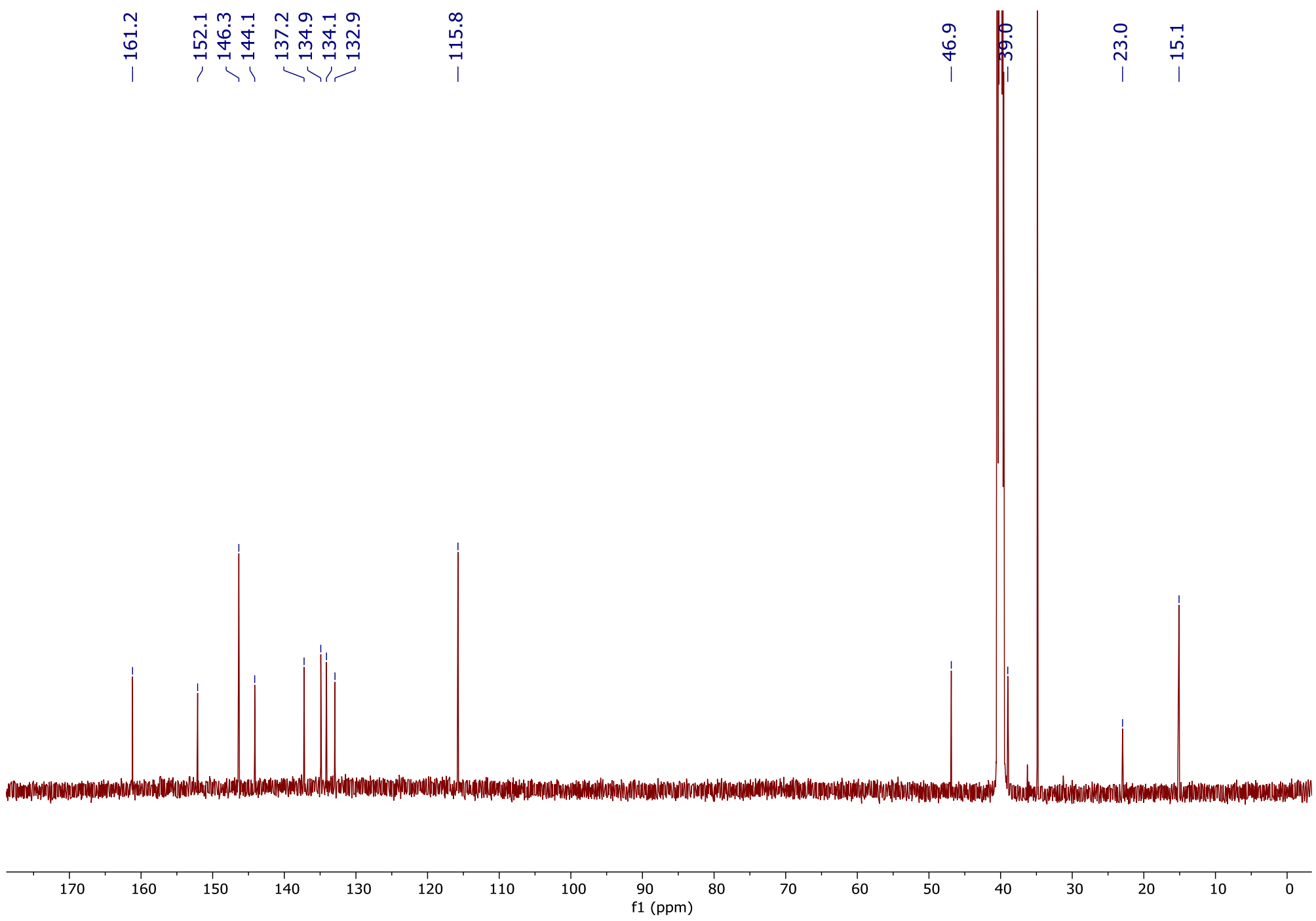

Figure S4. ^{13}C NMR spectrum of **2o**

Determination of the nature of the PSS.

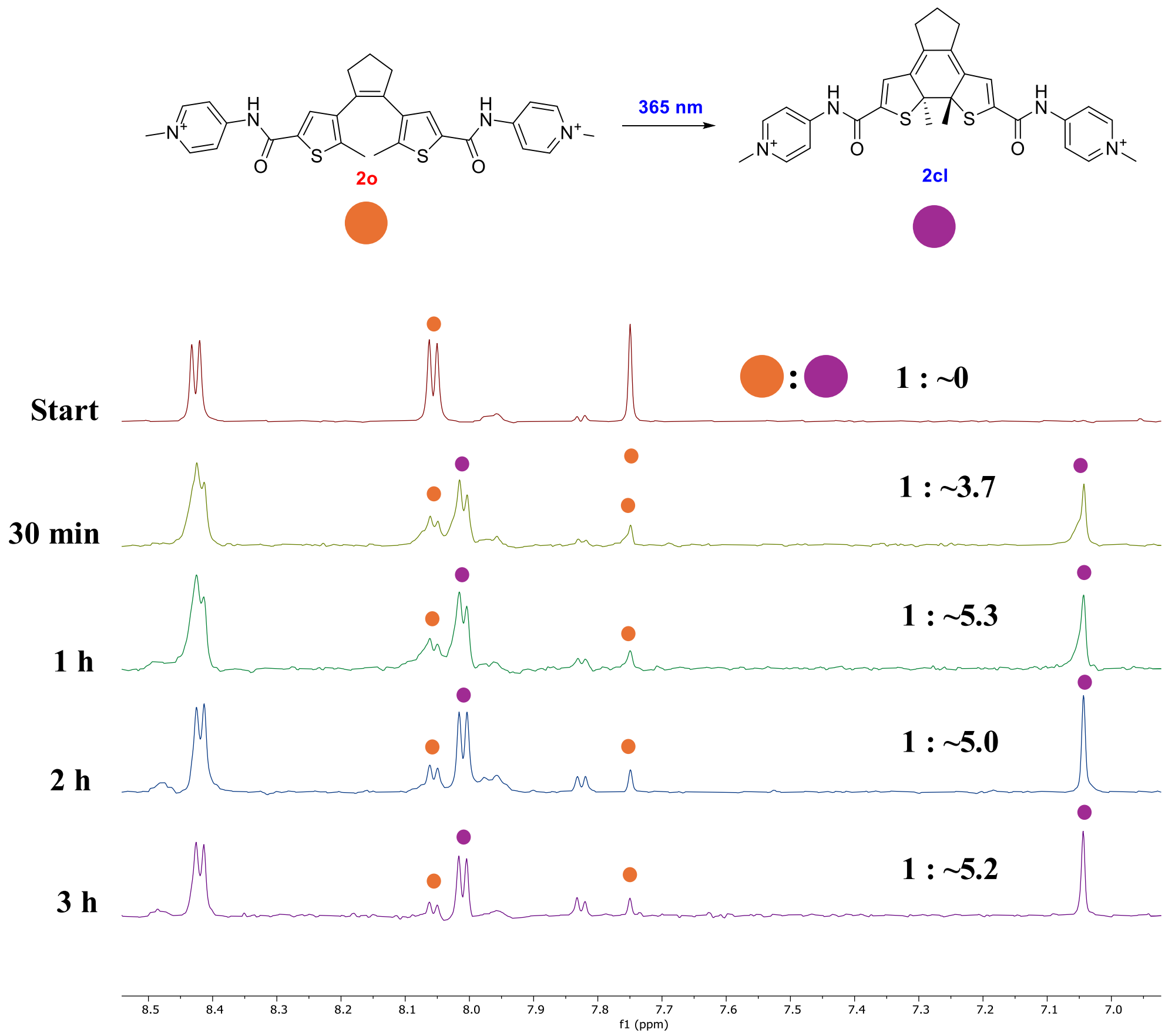

Figure S5. ^{1}H NMR spectra of **2o** under 365 nm irradiation (500 µM KPhos + 10% D_2O), with reduction of the peaks associated with **2o** (orange) decreasing as peaks associated with **2cl** (purple) increase. After around 1 h, there is no further increase in the ratio of **2cl**:**2o**, and the PSS was determined to be a 1:5 ratio of **2o**:**2cl**.

Thermal relaxation of the PSS at room temperature:

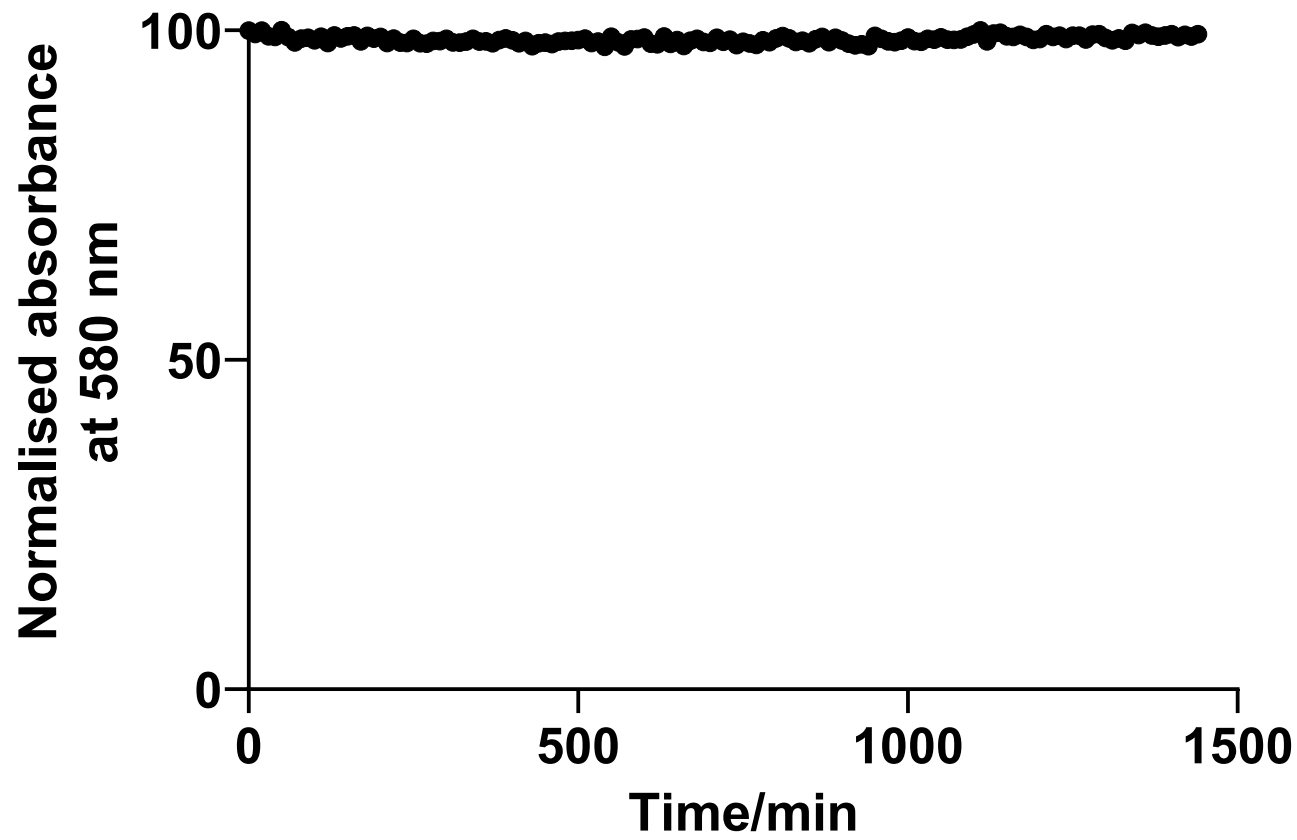

Figure S6. Normalised absorbance at 580 nm recorded by UV-Visible spectroscopy over time, monitoring any thermal relaxation of the PSS (100 µM KPhos) at room temperature in the dark. Minimal relaxation was observed up to 48 h under these conditions.

Thermal degradation of 2o:

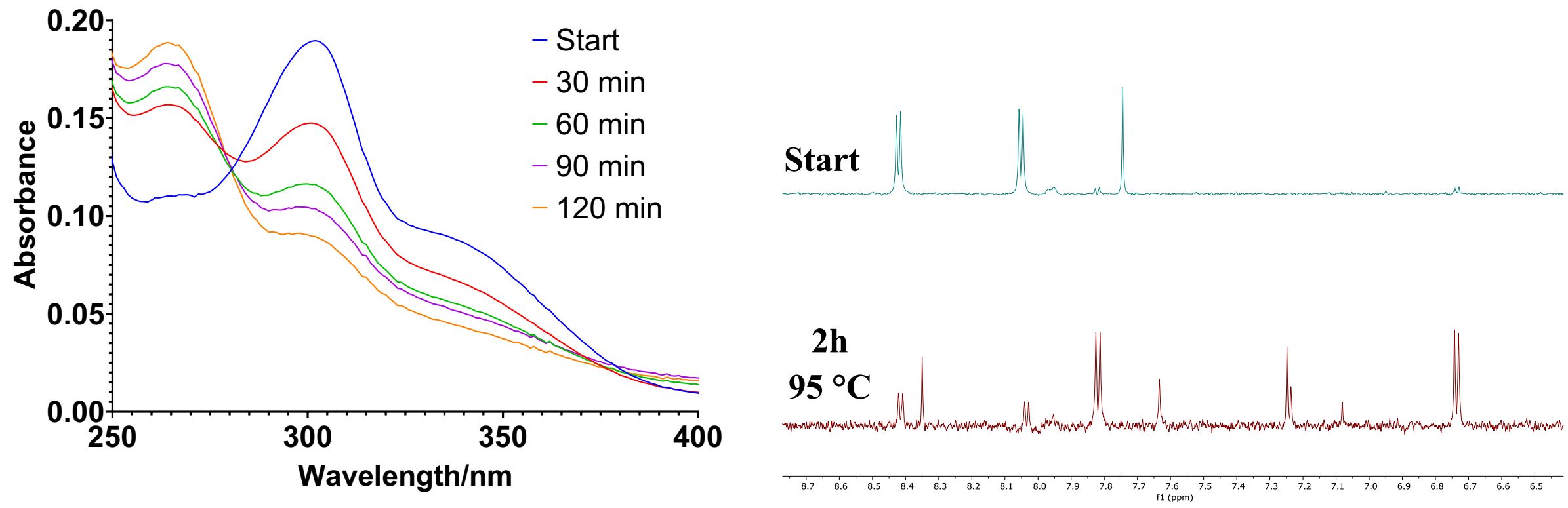

Figure S7. Left: UV-Visible spectrum of **2o** (100 µM KPhos) upon heating at 95 °C for up to 2 h in the dark. Right: ^{1}H NMR spectra (90 µM KPhos + 10% D_2O) for **2o** before and after heating to 95 °C for to 2 h in the dark, showing thermal degradation.

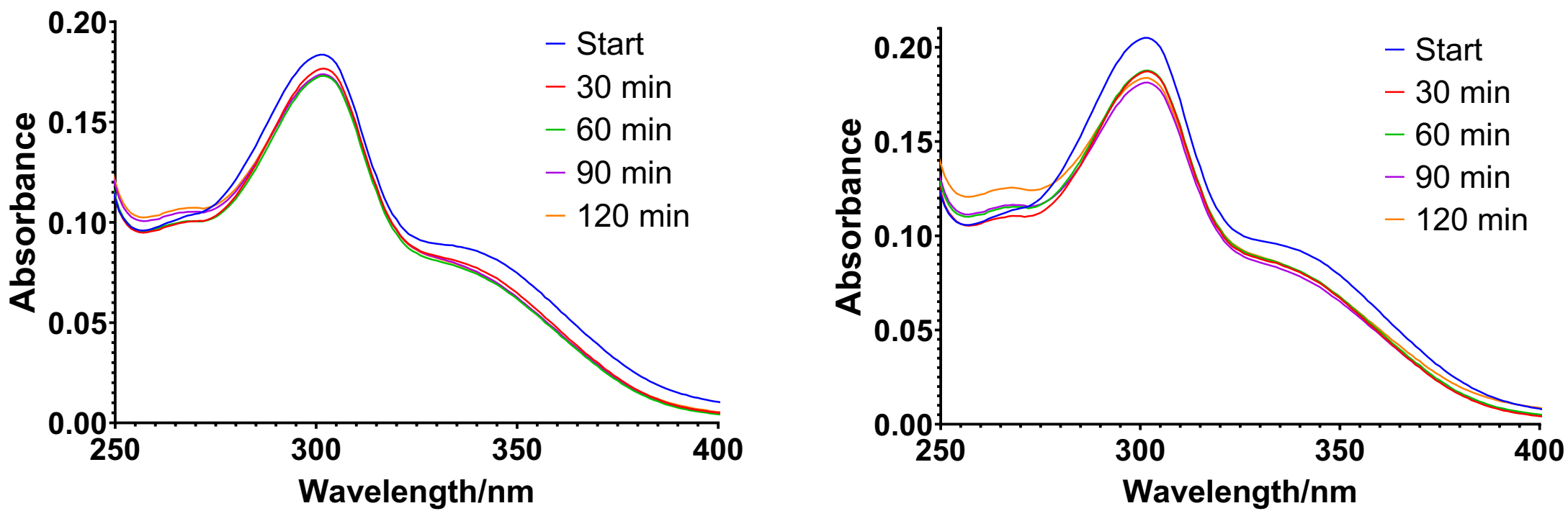

Figure S8. Left: UV-Visible spectrum of **2o** (100 µM KPhos) upon heating at 40 °C for up to 2 h in the dark. Right: UV-Visible spectrum of **2o** (100 µM KPhos) upon heating at 60 °C for up to 2 h in the dark.

Thermal degradation of PSS:

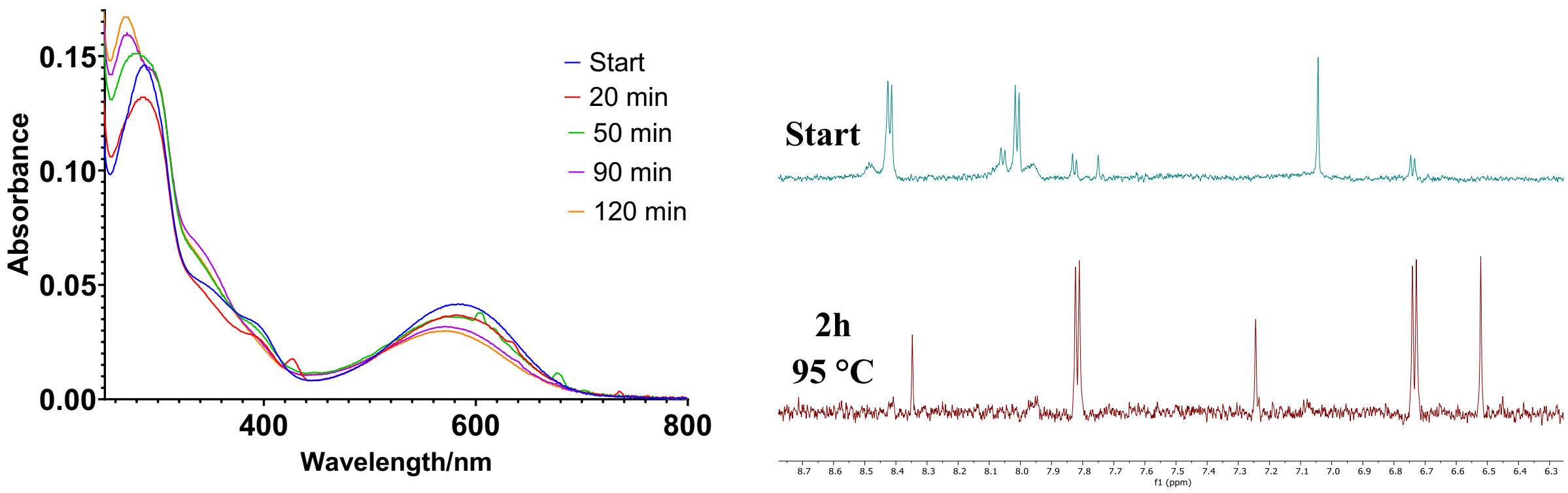

Figure S9. Left: UV-Visible spectrum of the PSS (100 µM KPhos) upon heating at 95 °C for up to 2 h in the dark. Right: ^{1}H NMR spectra (90 µM KPhos + 10% D_2O) for the **PSS** before and after heating to 95 °C for to 2 h in the dark, showing thermal degradation.

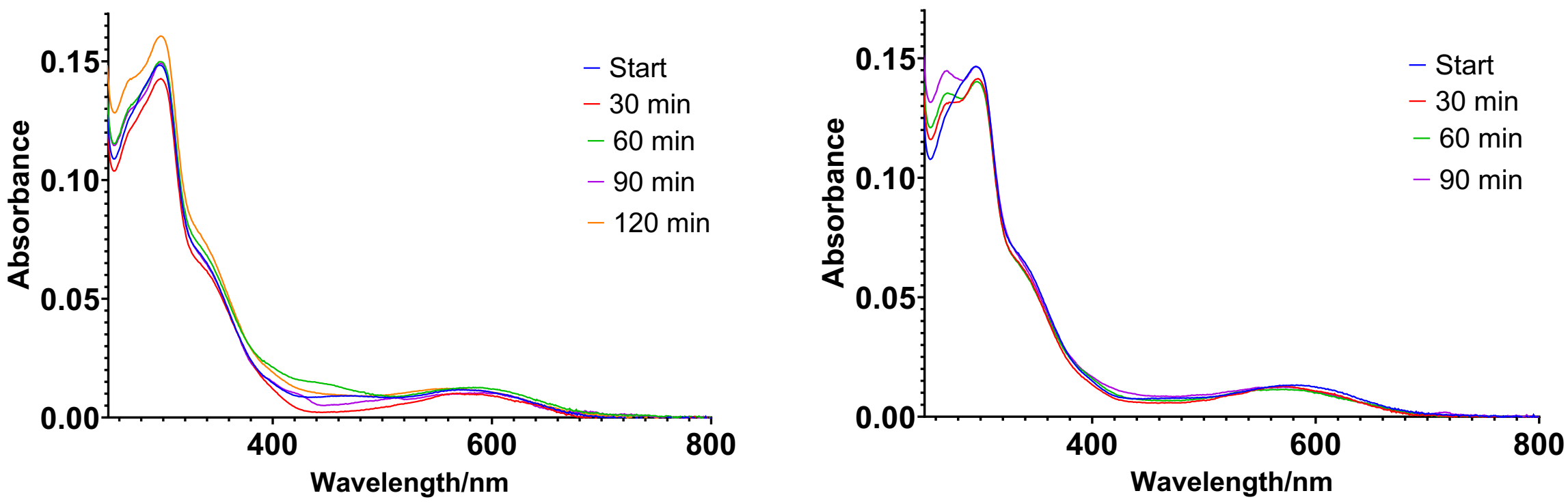

Figure S10. Left: UV-Visible spectrum of the **PSS** (100 µM KPhos) upon heating at 40 °C for up to 2 h in the dark. Right: UV-Visible spectrum of the **PSS** (100 µM KPhos) upon heating at 60 °C for up to 90 min in the dark.

Nucleic Acid Binding Studies

Materials used

Buffer composition:

K^+ buffer: 10 mM KCl, 90 mM LiCl and 10 mM Li cacodylate.

Na^+ buffer: 100 mM NaCl, and 10 mM Li cacodylate.

Low K^+ buffer: 1 mM KCl, 99 mM LiCl and 10 mM Li cacodylate

High K^+ buffer: 100 mM KCl and 10 mM Li cacodylate.

KPhos: 100 mM potassium phosphate, pH 7.4.

NaPhos: 100 mM sodium phosphate, pH 7.4.

FAM-TAMRA labelled nucleic acid sequences used for FRET melting assays:

Name	Sequence	Buffer, topology
F21T	5’-FAM-GGGTTAGGGTTAGGGTTAGGG-TAMRA-3’	K^+, h G4; Na^+, ap G4
F10T	5’-FAM-TATAGCTATA-HEG-TATAGCTATA-TAMRA-3’	K^+, duplex
FMycT	5’-FAM-TTGAGGGTGGGTAGGGTGGGTAA-TAMRA-3’	Low K^+, p G4

FAM = fluorescein dye, TAMRA = rhodamine dye, HEG = [$(-CH_2CH_2O-)_6$], h = hybrid, ap = antiparallel, p = parallel.

Non-fluorescently labelled DNA sequences:

Name	Sequence	Buffer, topology
Telo23	5’-TAGGGTTAGGGTTAGGGTTAGGG-3’	KPhos, h G4
Telo22	5’-AGGGTTAGGGTTAGGGTTAGGG-3’	NaPhos ap G4
c-Myc	5’-TTGAGGGTGGGTAGGGTGGGTAA-3’	KPhos, p G4
Ds26	5'-CAATCGGATCGAATTCGATCCGATTG-3'	KPhos, duplex

FRET Melting Assays.

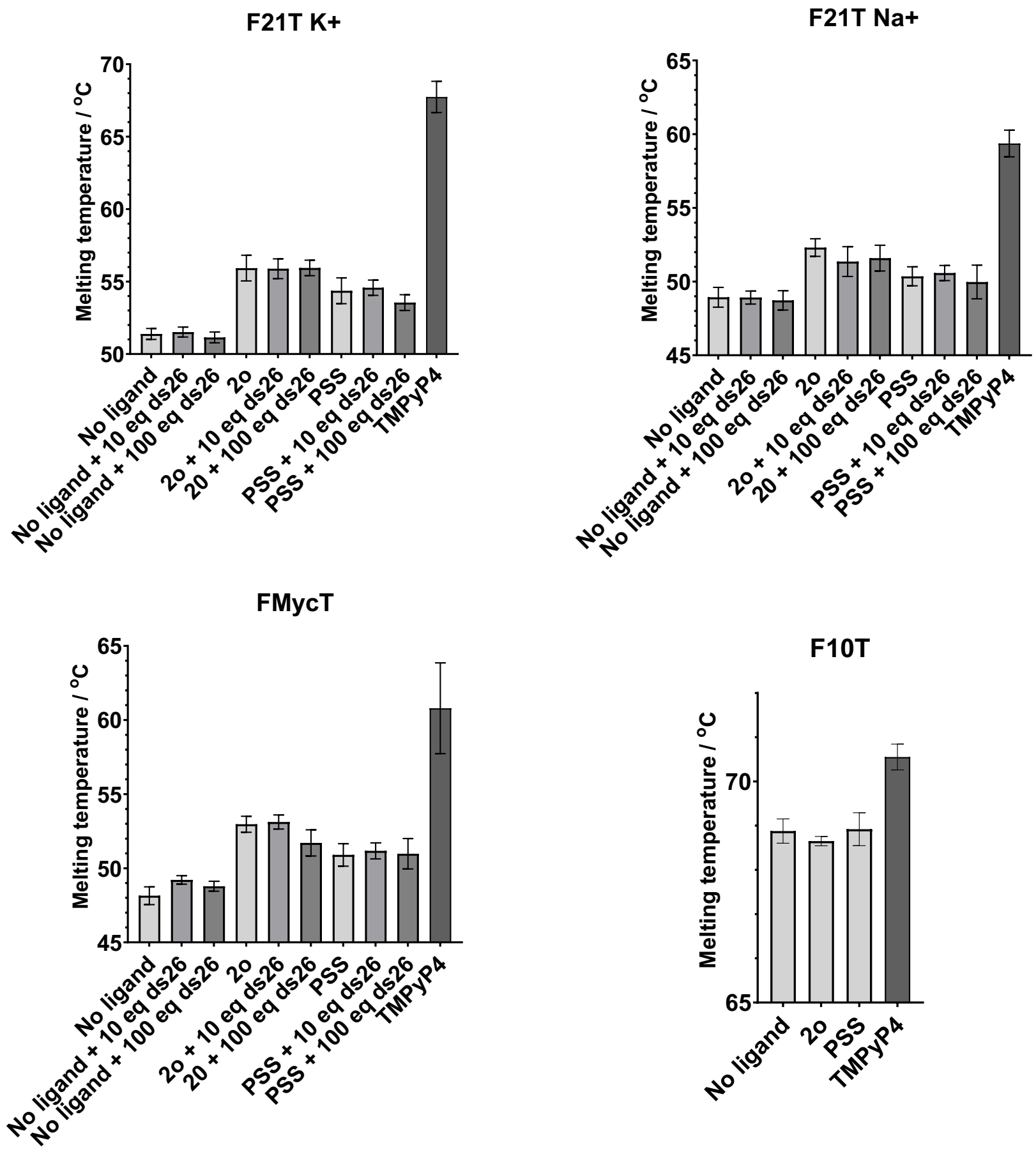

Figure S11. Melting temperatures for F21T K$^+$ (top left), F21T Na+ (top right), FMycT (bottom left) and F10T (bottom right), with either HPLC water (no ligand), 1 µM TMPyP4 or 10 µM of **2o** or the **PSS**, with increasing concentrations of duplex DNA (10 or 100 eq of ds26).

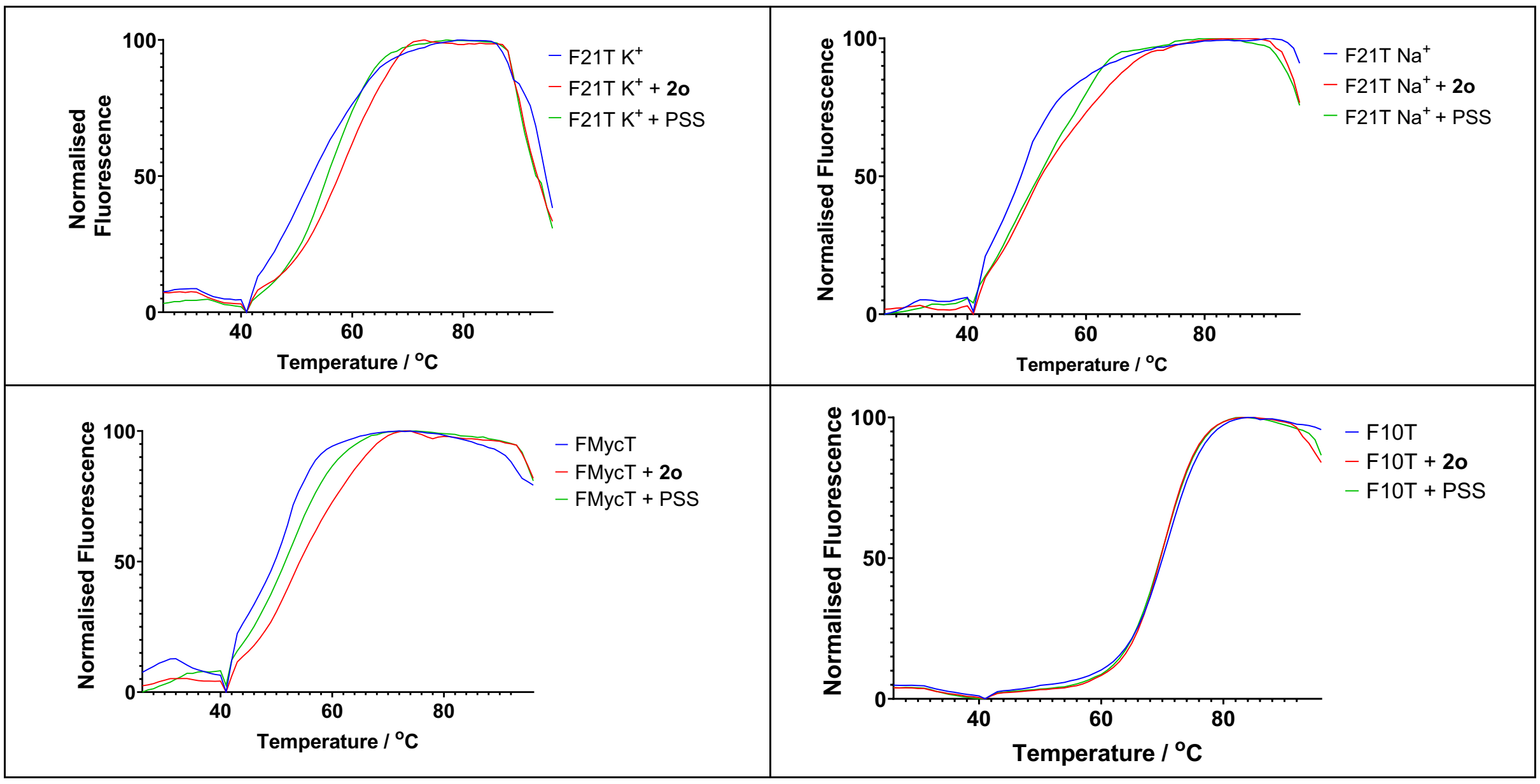

Figure S12. Example melting profiles for each of the DNA sequences tested. A single representative melting profile (of 8 replicates) is provided for F21T K$^+$ (top left), F21T Na+ (top right), FMycT (bottom left) and F10T (bottom right), with HPLC water (no ligand), 10 µM of **2o** and the **PSS**.

CD spectroscopy titrations.

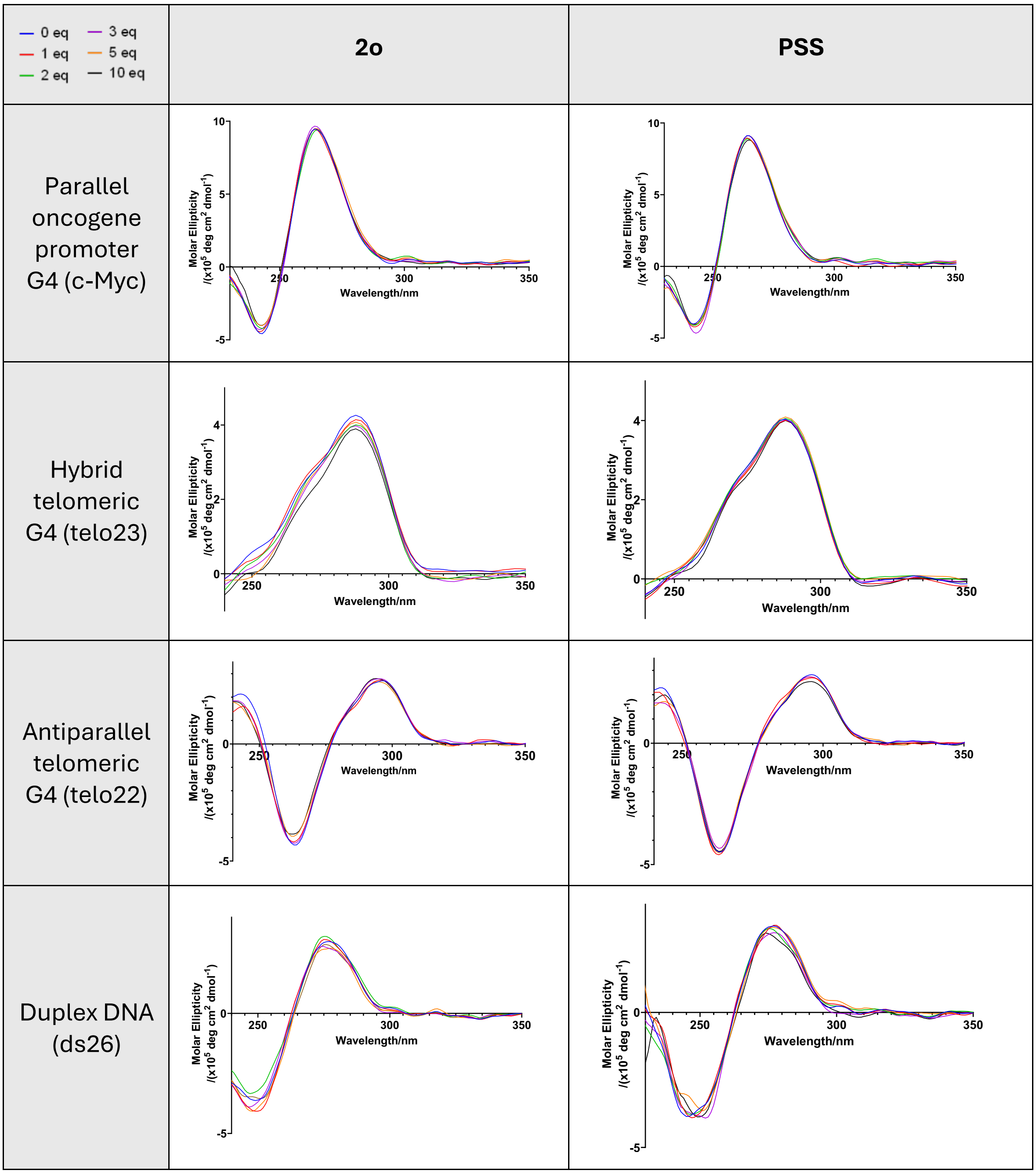

Figure S13. Observed spectral changes on addition of up to 10 eq of **2o** or the PSS observed by CD spectroscopy.

UV-Visible spectroscopy titrations.

The binding model used to analyse the data was a multiple independent binding site model – as described by P. Thordarson – and K_a was calculated in GraphPad Prism according to the following equation:[1]

$$\Delta A = \varepsilon_{DNA.Ligand} \frac{\left(N[DNA] + [Ligand] + \frac{1}{K_a}\right) + \sqrt{\left(N[DNA] + [Ligand] + \frac{1}{K_a}\right)^2 - 4N[DNA][Ligand]}}{2}$$

ΔA = Change in UV-vis aborbance, $\varepsilon_{DNA.Ligand}$ = extinction coefficient for DNA:ligand complex, N = number of binding sites (in this case assuming N=1 gave best fit), [DNA] = concentration of DNA, [ligand] = concentration of ligand, K_a = association constant for DNA:ligand interaction.

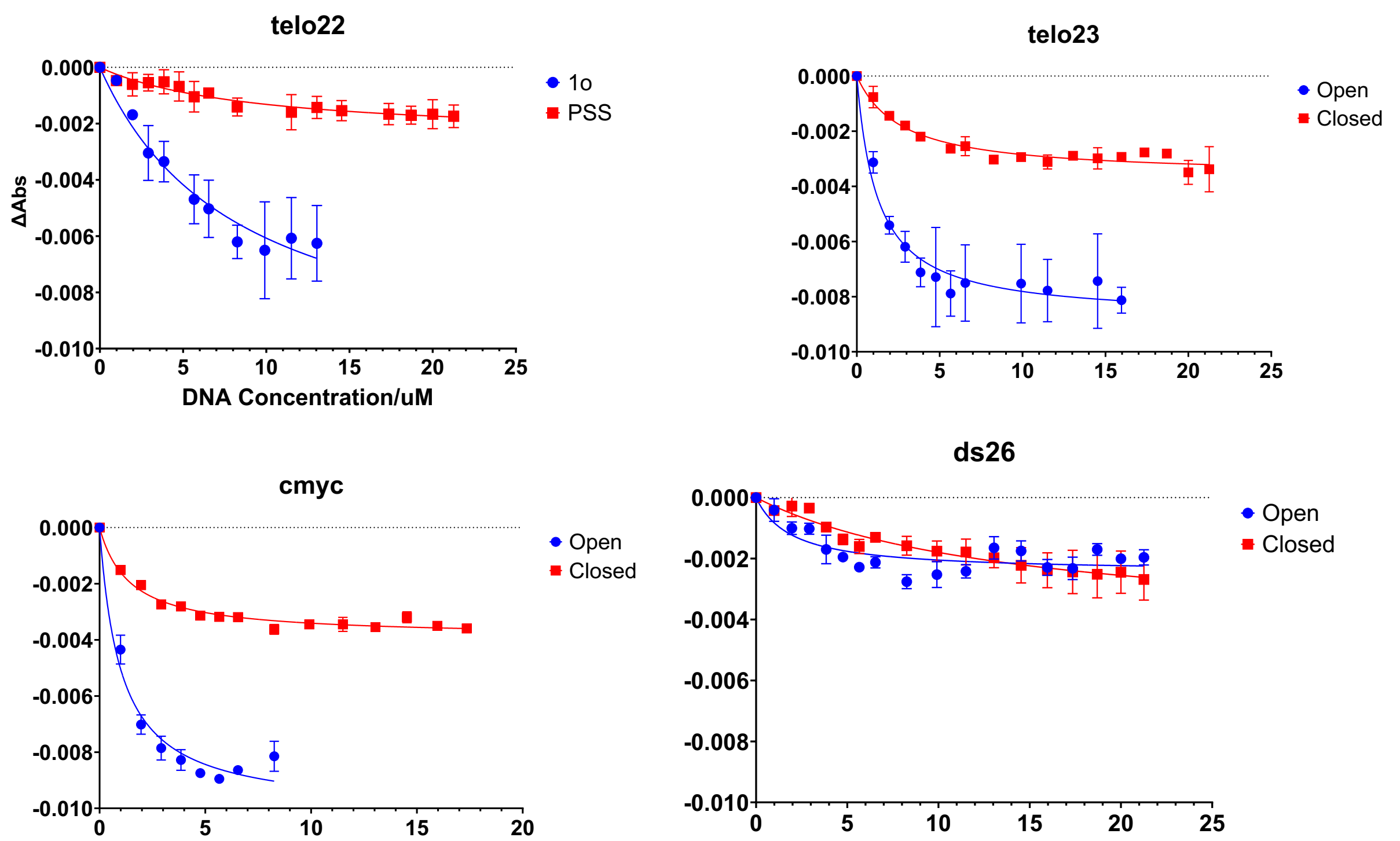

Figure S14. Change in UV-Visible absorbance at the wavelength of maximal change (340 nm for **2o**, 580 nm for **PSS**) on increasing concentration of telo22 (top left), telo23 (top right), c-Myc (bottom left) or ds26 (bottom right). Non-linear regression curves used to determine the binding affinities are overlaid over the raw datapoints.

NMR spectroscopy titrations.

Assignments used

Telo22:

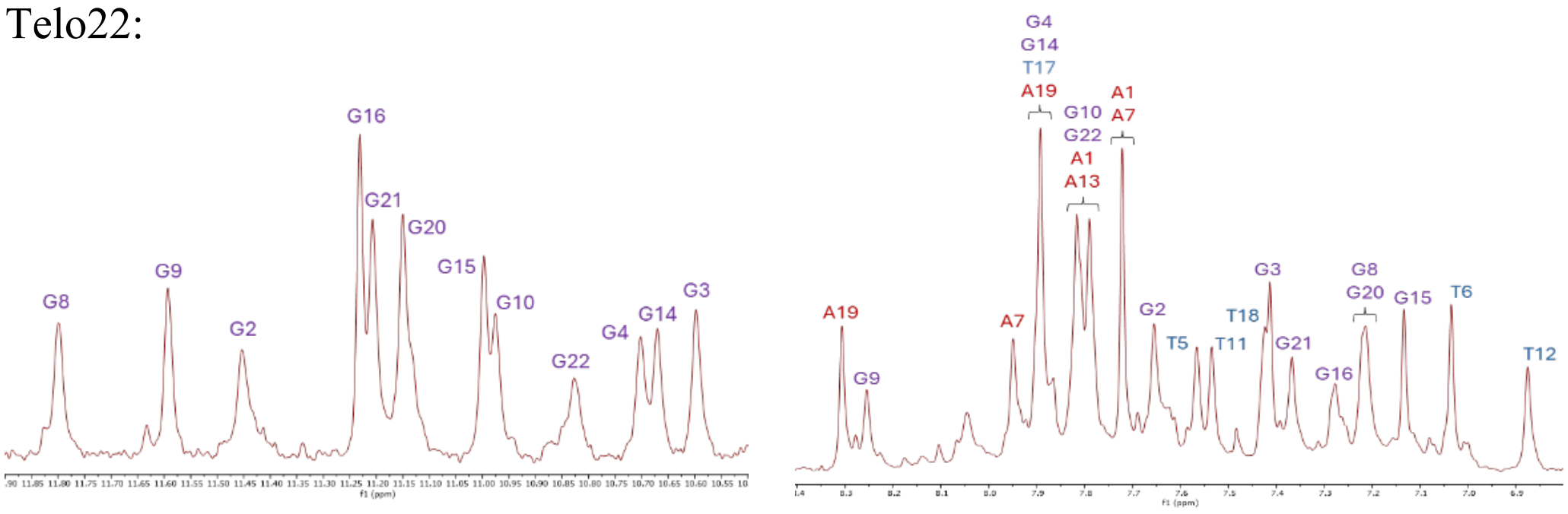

Figure S15. Assignments for telo22 in NaPhos, assigned with reference spectra from the literature.[2]

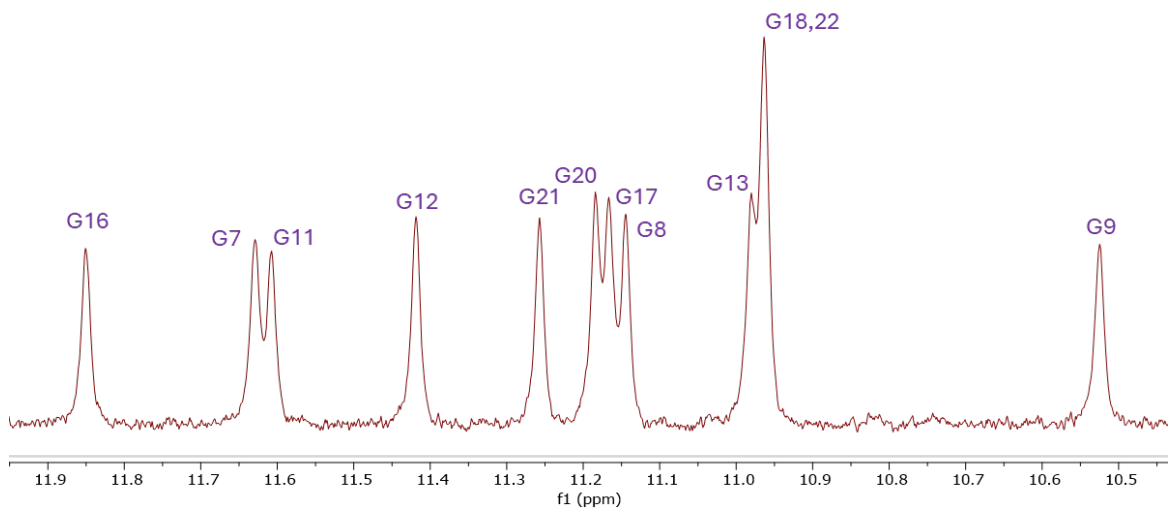

Figure S16. Assignments for c-Myc in KPhos, assigned with reference spectra from the literature.[3]

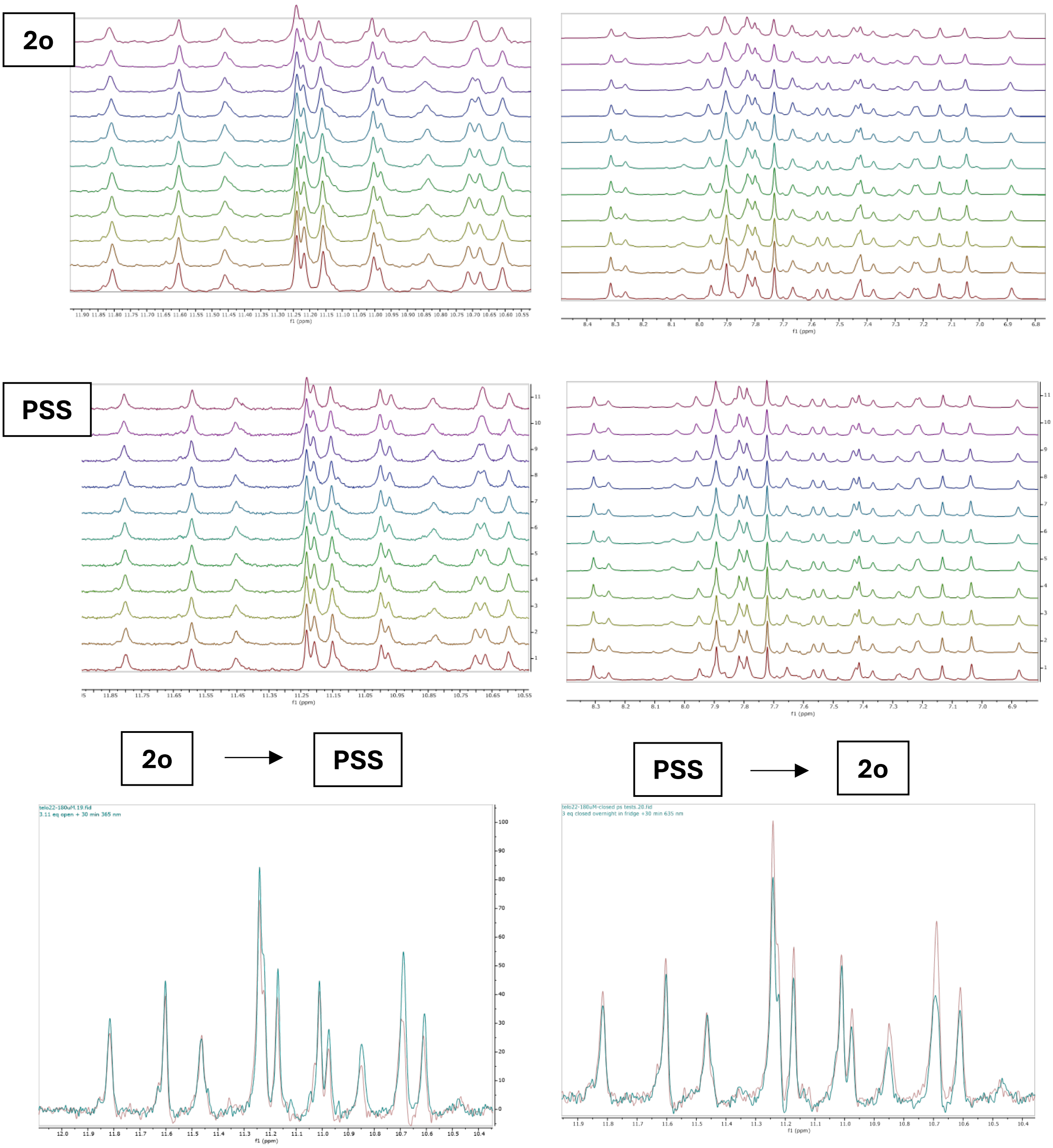

Figure S17. ^{1}H NMR spectra for telo22 titrations with increasing concentrations of **2o** (top section), PSS (middle section) or upon light irradiation after 3 eq of ligand added to interconvert between the photo-isomers from **2o** to **PSS** and **PSS** to **2o** (bottom section).

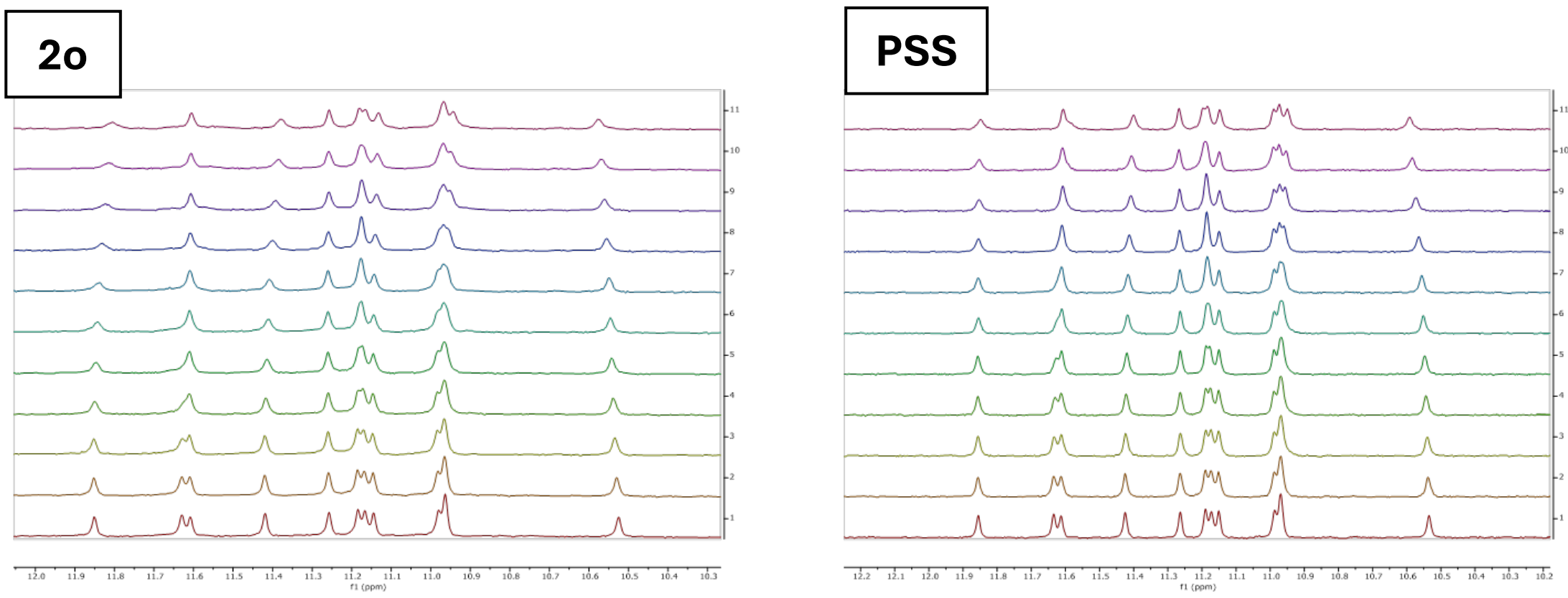

Figure S18. ^{1}H NMR spectra for c-Myc titrations with increasing concentrations of **2o** (left), **PSS** (right).

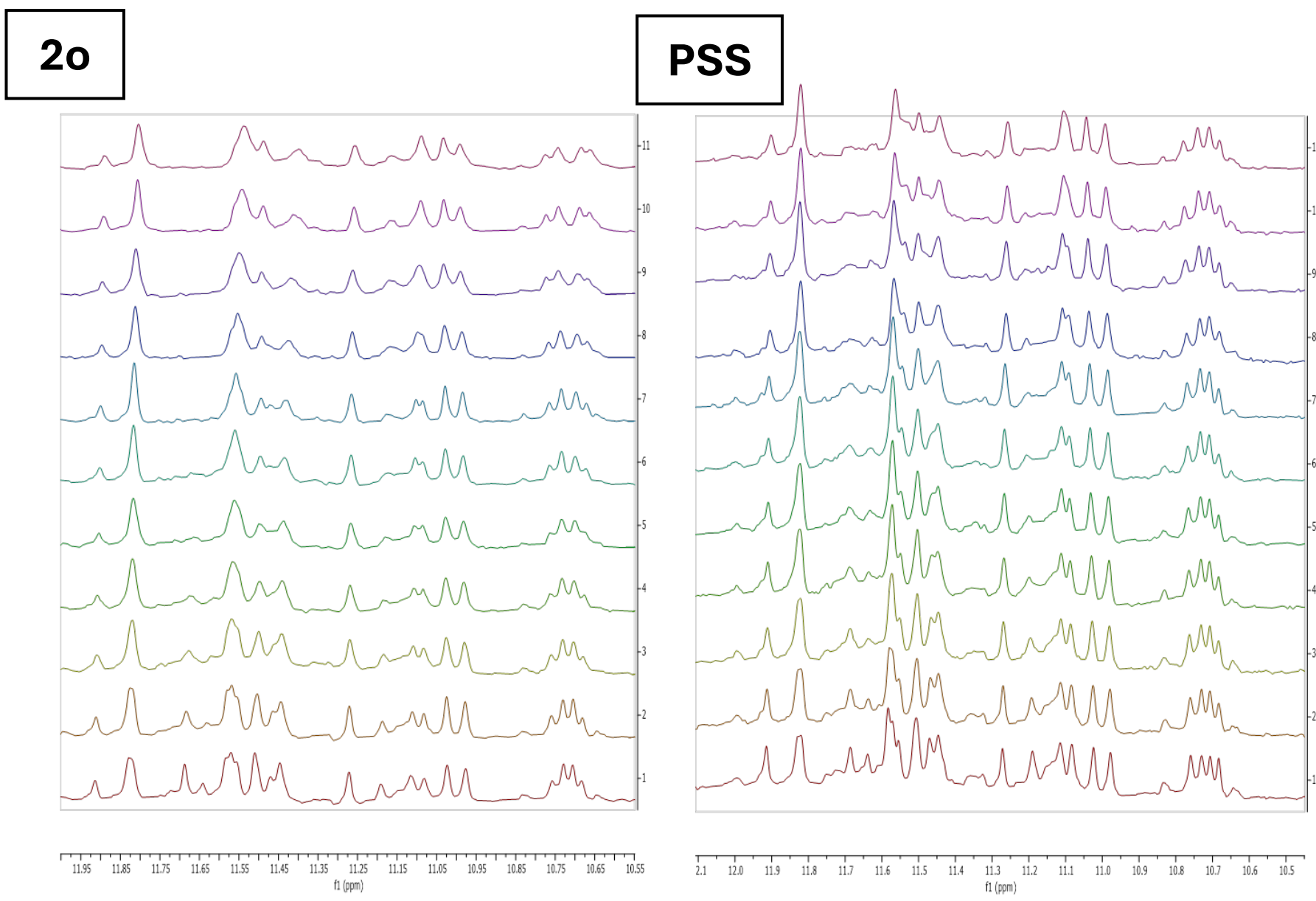

Figure S19. ^{1}H NMR spectra for telo23 titrations with increasing concentrations of **2o** (left), PSS (right).

Molecular Docking Calculations

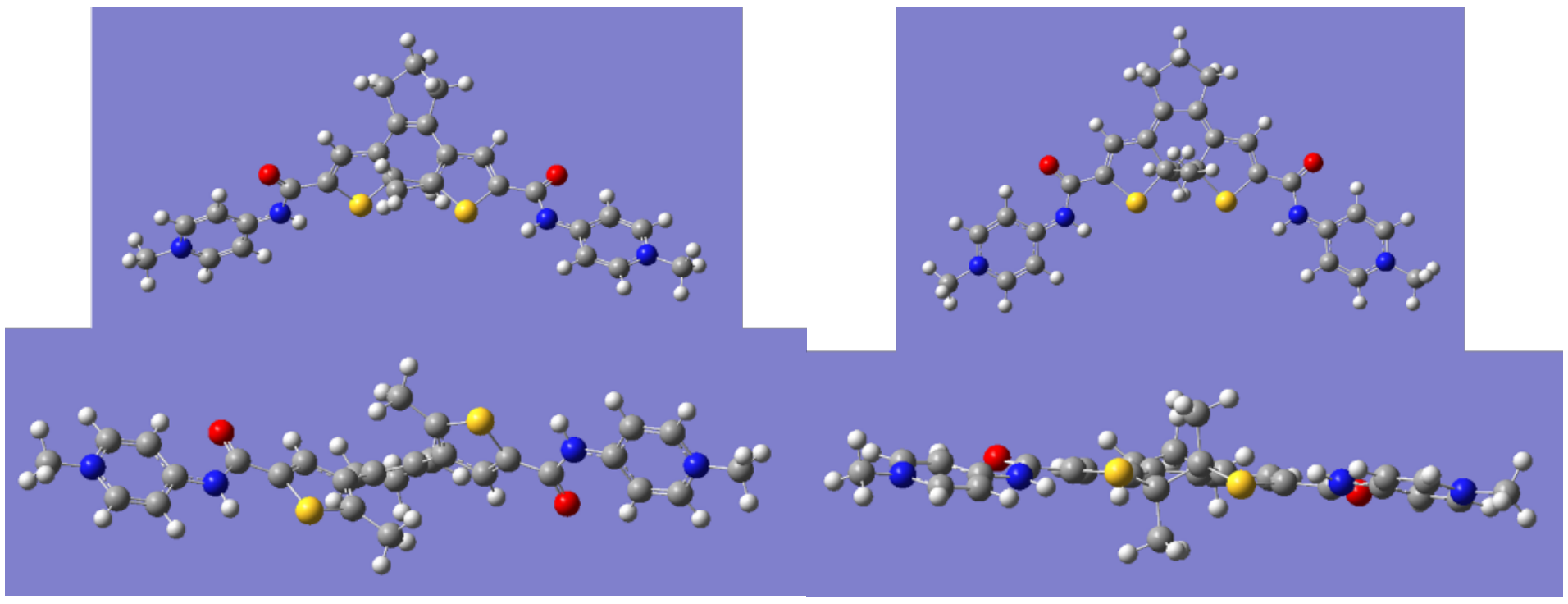

Figure S20. Geometry-optimized structures of **2o** (open, left) and **2cl** (closed, right). These structures were obtained after a geometry optimization using Gaussian 16[4] with a B3LYP functional and the 6-31g (d) basis set. The white, blue, red, yellow and grey spheres represent hydrogen, nitrogen, oxygen, sulfur and carbon atoms, respectively.

For telo22, both photo-isomers gave a range of potential groove-binding interactions (Figure 6, ESI figures 21 and 22). For the open-isomer, the most stable predicted binding modes were in the rear or right-hand groove, with the bent-shape and flexibility of the ligand allowing for interactions with the groove and loop nucleotides. Minimal evidence of face-face pi-stacking was observed in some of the models (with A7 in the top-right loop), with most interactions from end-face and offset pi-stacking (with A7 and A13 in the loops, G8-10, G20-22 on the rear face and G3-4 and G8-9 on the right face) and interactions with the sugar-phosphate backbone of the groove and loop nucleotides. For the closed isomer, the most-stable predicted binding modes were in the right- or left-hand grooves, with the flatter and more rigid structure preferring to interact within a single groove rather than bending to interact with the loops. No evidence of face-to-face pi-stacking was observed in the models, with some end-face and offset pi-stacking (with A1, T11 and T12 in the loops, G14-16, G20-21 in the left groove, and G2-G4 and G8-G10 in the right groove) and interactions with the sugar-phosphate backbone primarily within the groove. The more peripheral interactions with this ligand may help explain the lower affinity and structural perturbations observed with the closed-form enriched PSS, while the flexibility of the open form allows for tighter and more substantial interactions. From these models, the increased flexibility of the extended amide linkage in **2** appears to allow for stable groove-binding interactions with the antiparallel structure. Additionally, the H-bonding capability of the amide may also contribute to the significant sugar-phosphate backbone interactions observed, which could help explain the increased affinity of **2** compared to **1**.

Model	Energy (kcal/mol)	Model	Energy (kcal/mol)
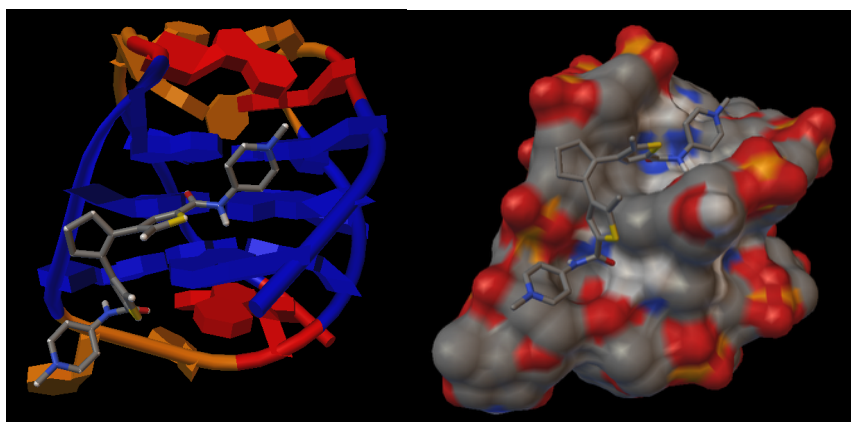	-7.8	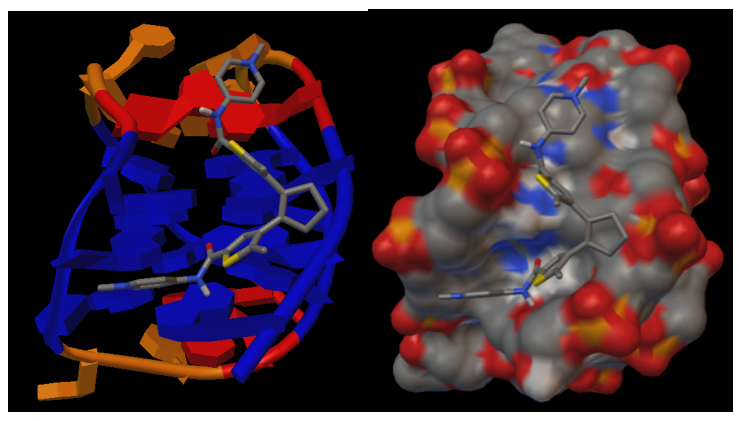	-7.4
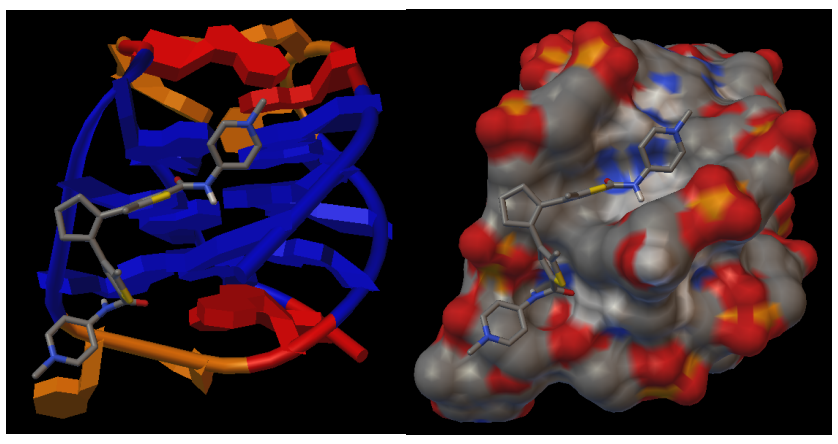	-7.8	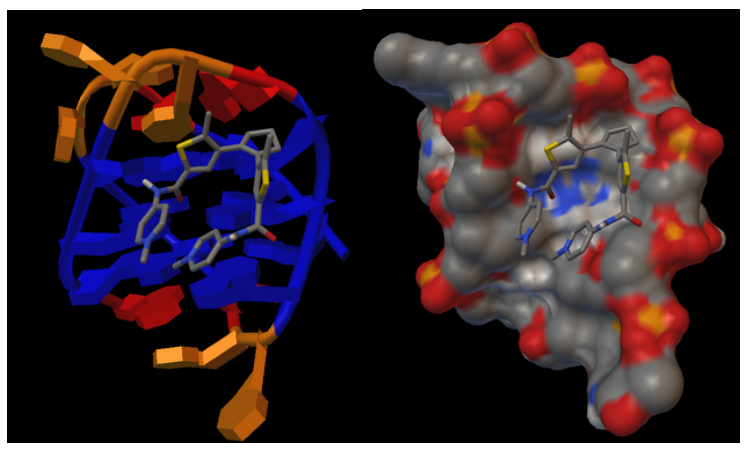	-7.4
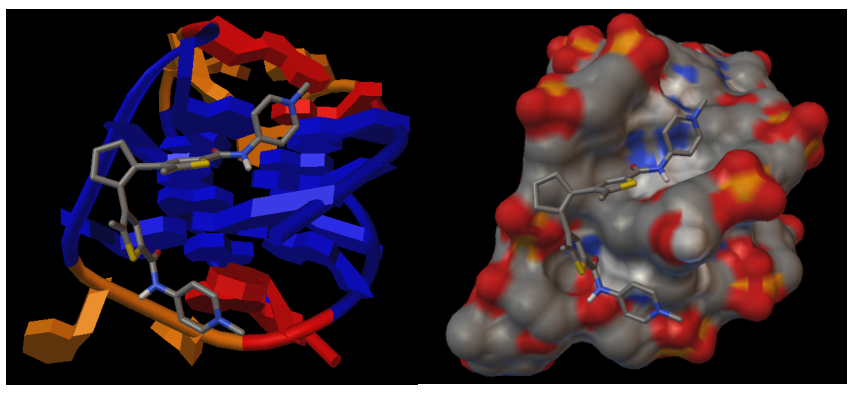	-7.7	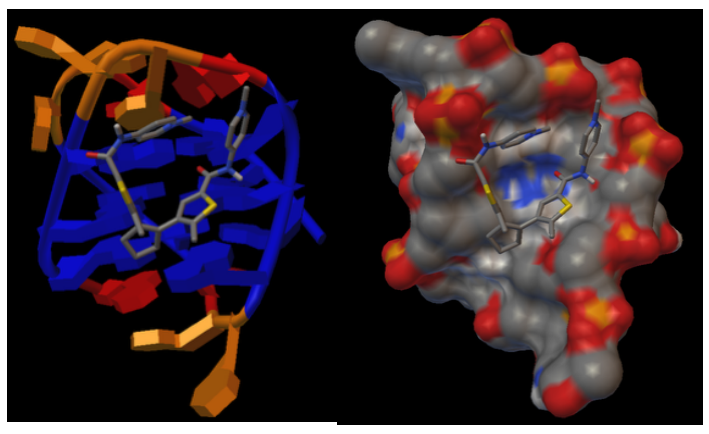	-7.4
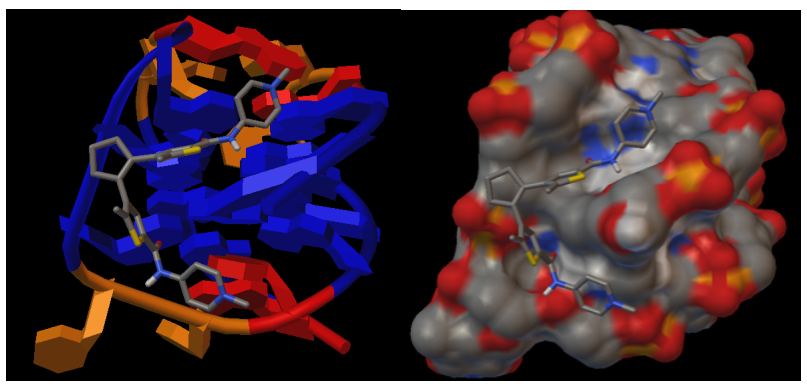	-7.6	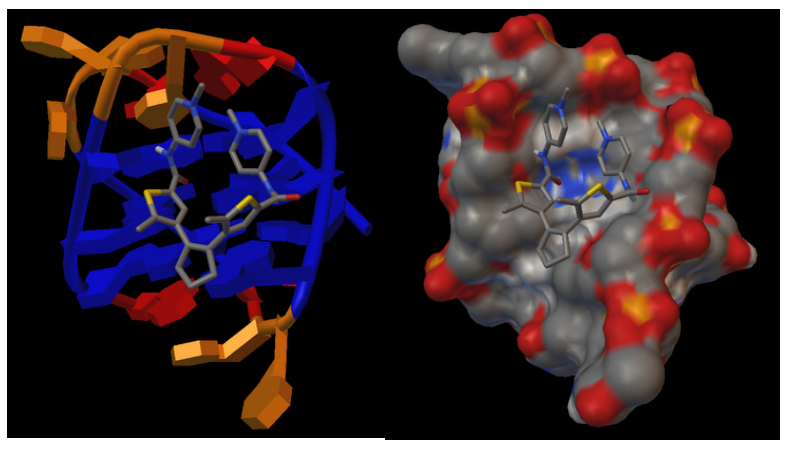	-7.4

Figure S21. The eight best-scoring (i.e. lowest-energy) poses for compound **2o** when bound to the antiparallel telo22 (PDB: 143D, 5'-AGGGTTAGGGTTAGGGTTAGGG-3') and corresponding binding energies. The docking calculations were performed using Autodock Vina.[5] Each model is shown as a cartoon colored by base (A = red, T = orange, G = blue) with an overlaid molecular surface colored by atom type (C = grey, N = blue, O = red, S = orange). The **2o** ligand is depicted using a stick representation.

Model	Energy (kcal/mol)	Model	Energy (kcal/mol)
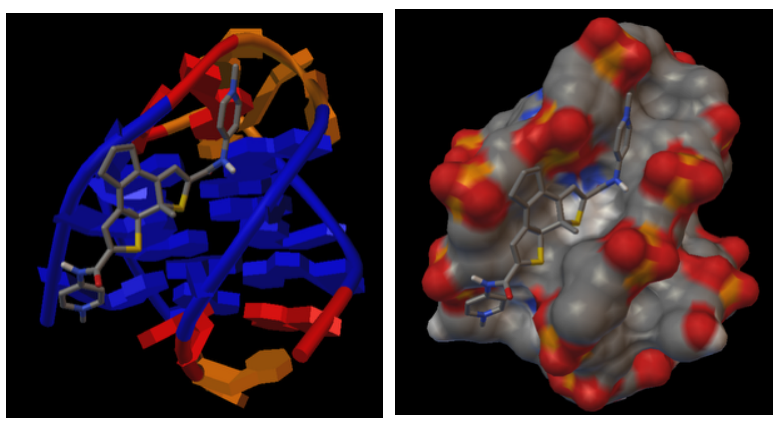	-7.5	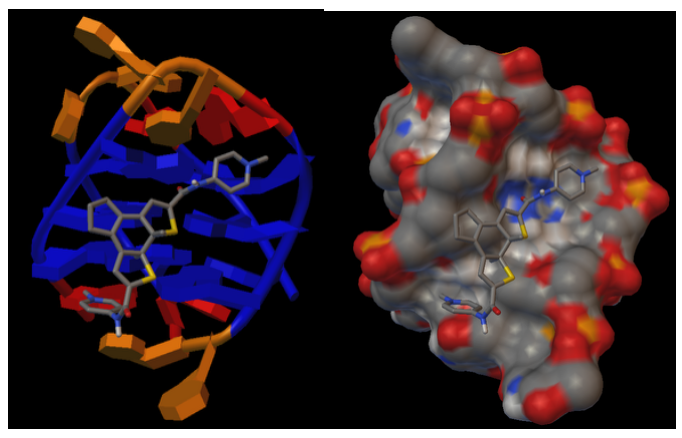	-7.3
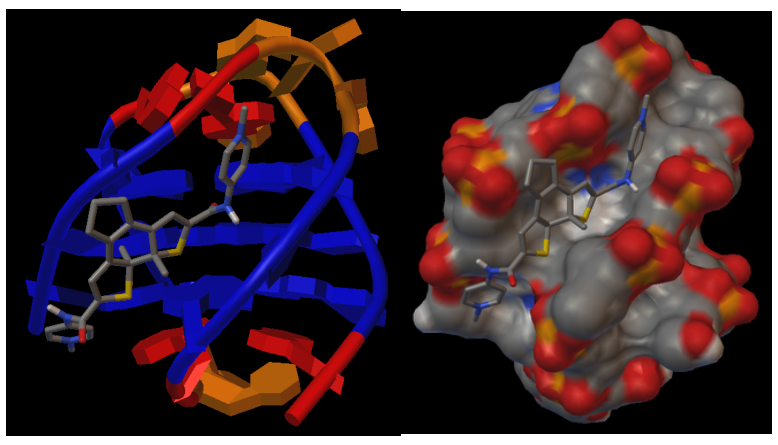	-7.5	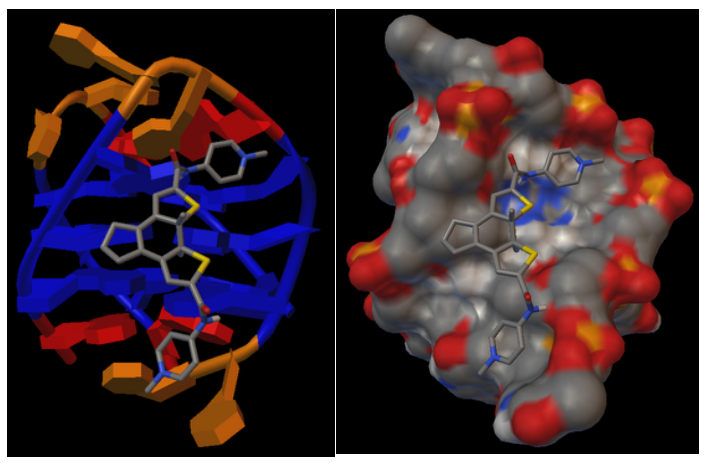	-7.3
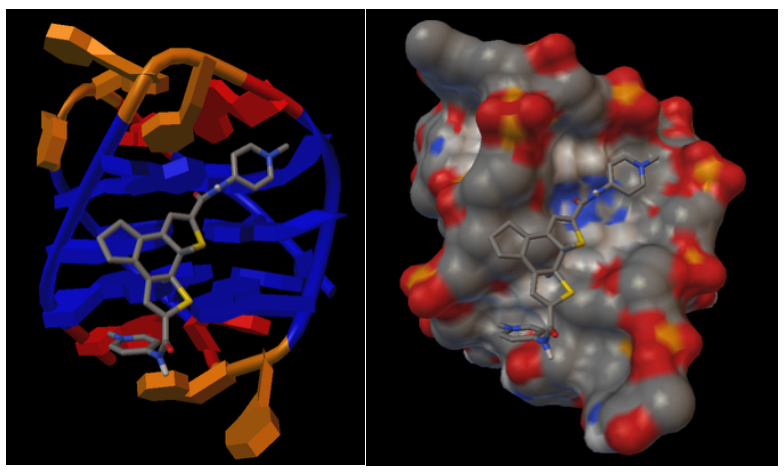	-7.4	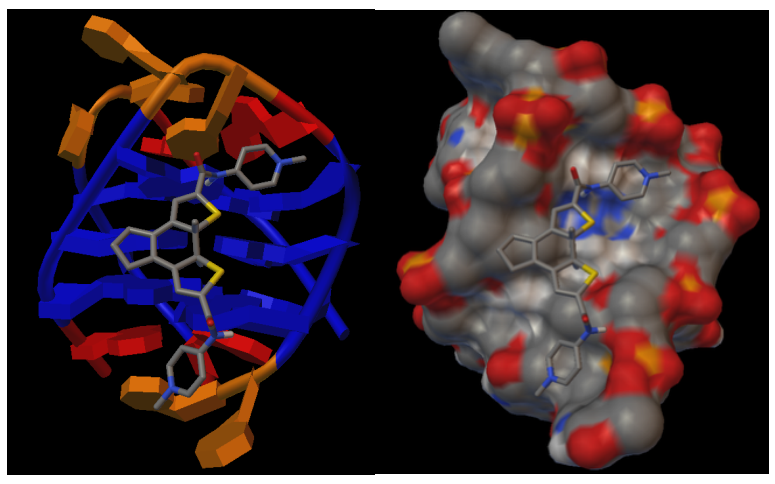	-7.2
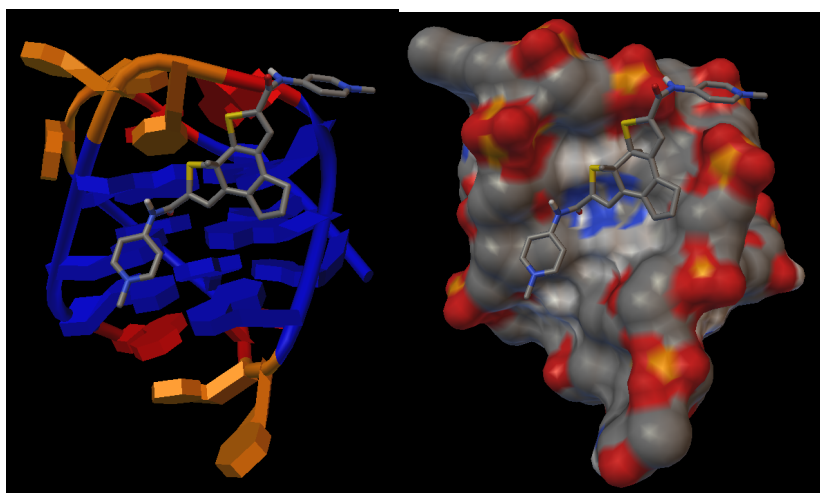	-7.4	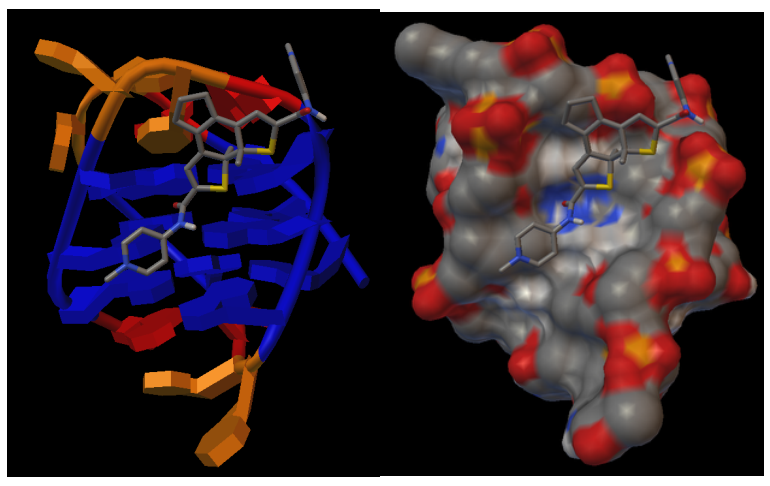	-7.2

Figure S22. The eight best-scoring (i.e. lowest-energy) poses for compound **2cl** when bound to the antiparallel telo22 (PDB: 143D, 5’-AGGGTTAGGGTTAGGGTTAGGG-3’) and corresponding binding energies. The docking calculations were performed using Autodock Vina.[5] Each model is shown as a cartoon colored by base (A = red, T = orange, G = blue) with an overlaid molecular surface colored by atom type (C = grey, N = blue, O = red, S = orange). The **2cl** ligand is depicted using a stick representation.

Model	Energy (kcal/mol)	Model	Energy (kcal/mol)
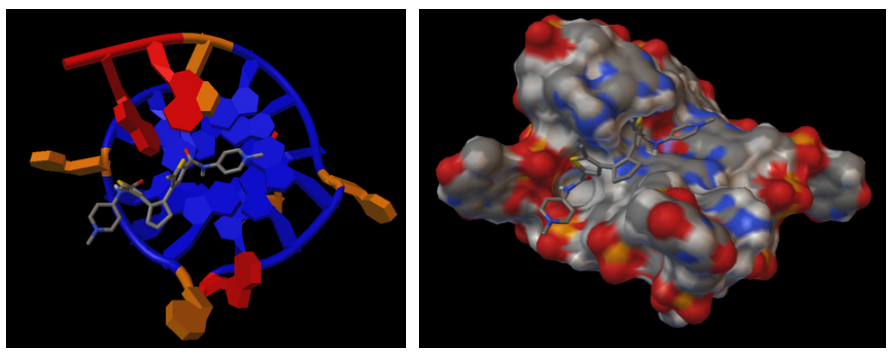	-7.9	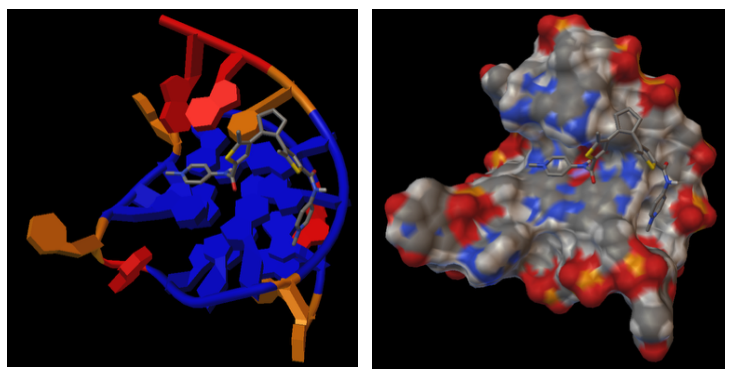	-7.7
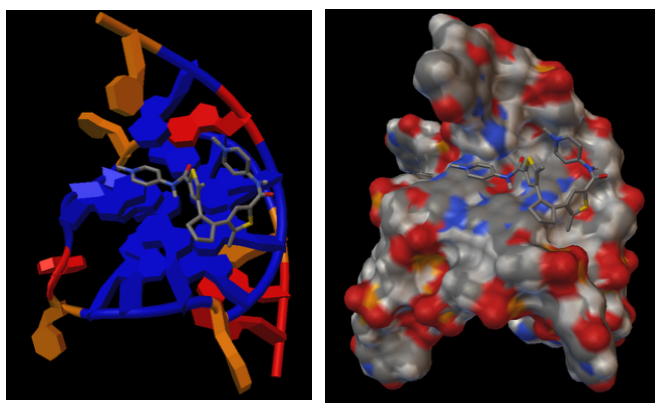	-7.8	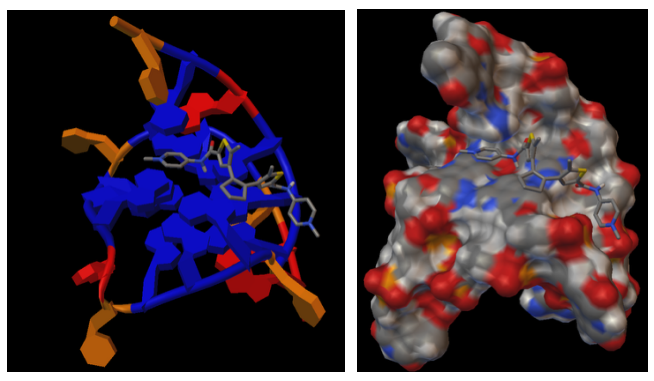	-7.7
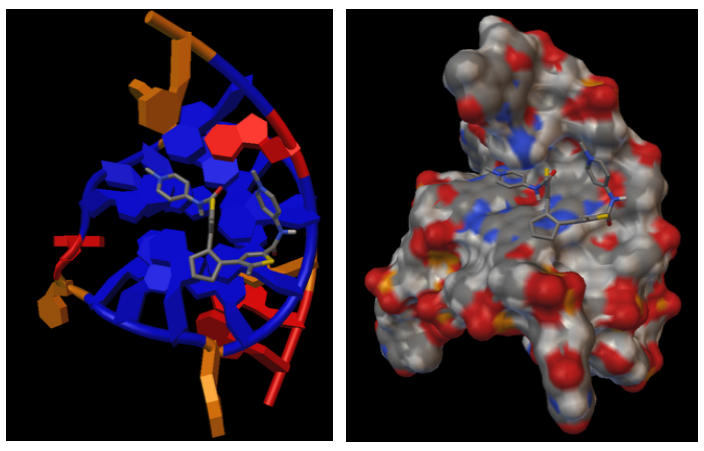	-7.7	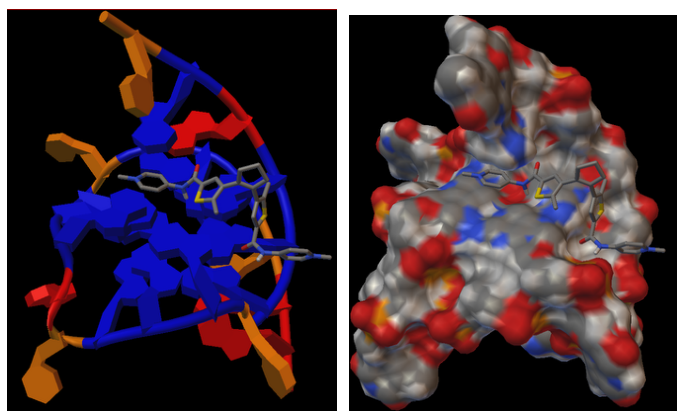	-7.7
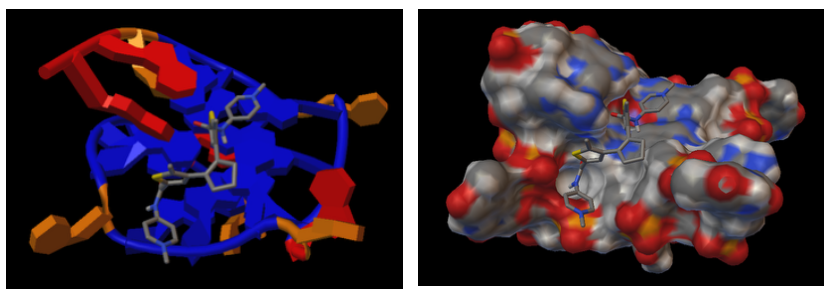	-7.7	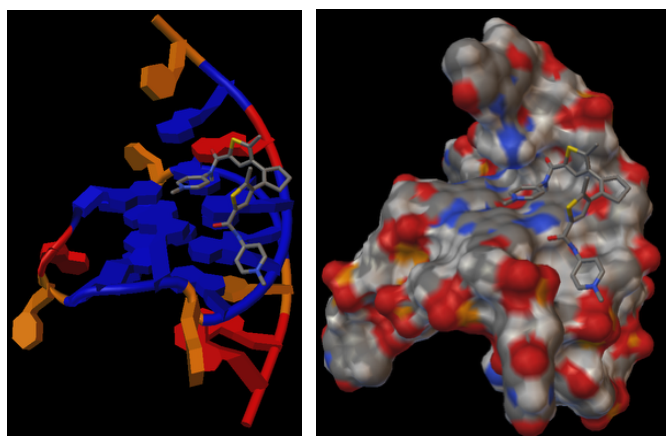	-7.6

Figure S23. The eight best-scoring (i.e. lowest-energy) poses for compound **2o** when bound to the parallel c-Myc (PDB: 2L7V, 5'-TGAGGGTGGGTAGGGTGGGTAA-3') and corresponding binding energies. The docking calculations were performed using Autodock Vina.[5] Each model is shown as a cartoon colored by base (A = red, T = orange, G = blue) with an overlaid molecular surface colored by atom type (C = grey, N = blue, O = red, S = orange). The **2o** ligand is depicted using a stick representation.

Model	Energy (kcal/mol)	Model	Energy (kcal/mol)
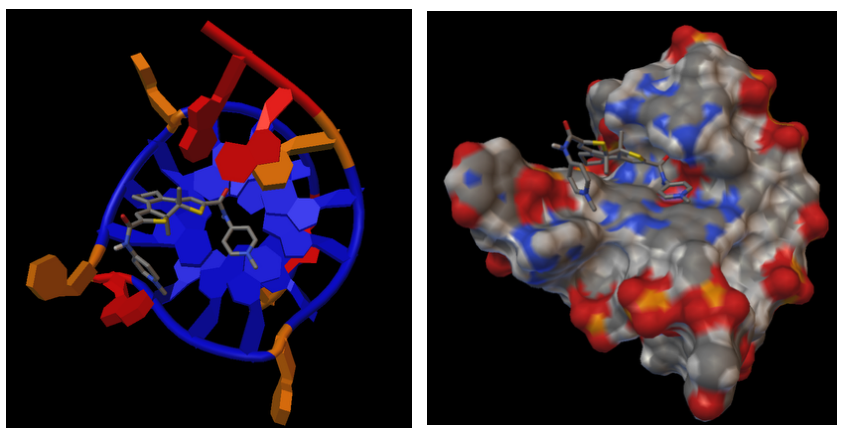	-7.5	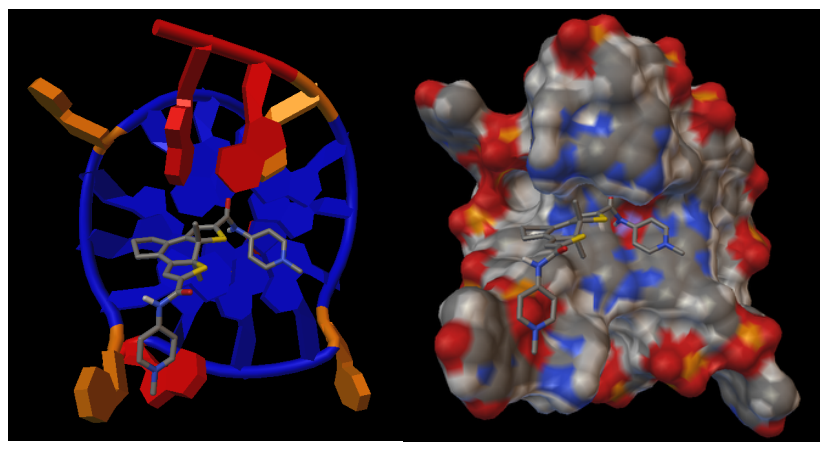	-7.4
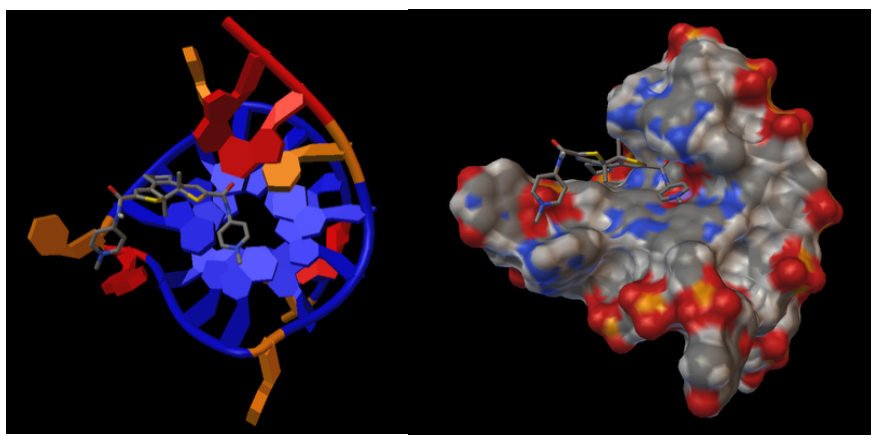	-7.4	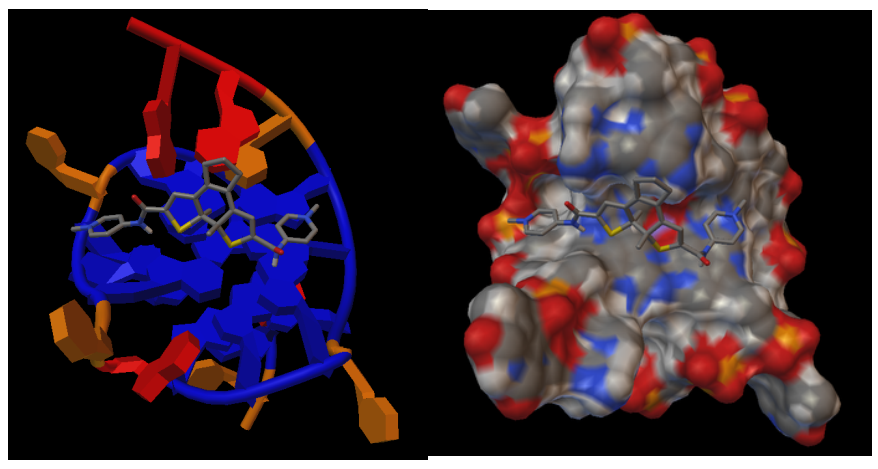	-7.3
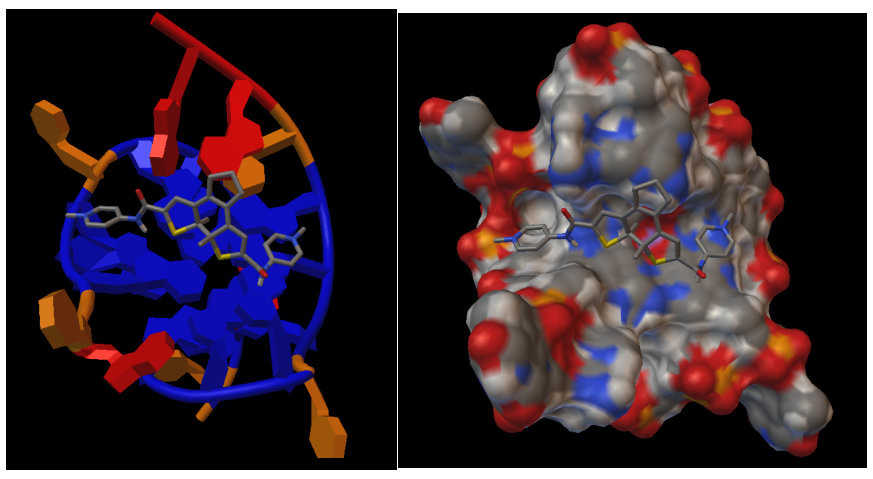	-7.4	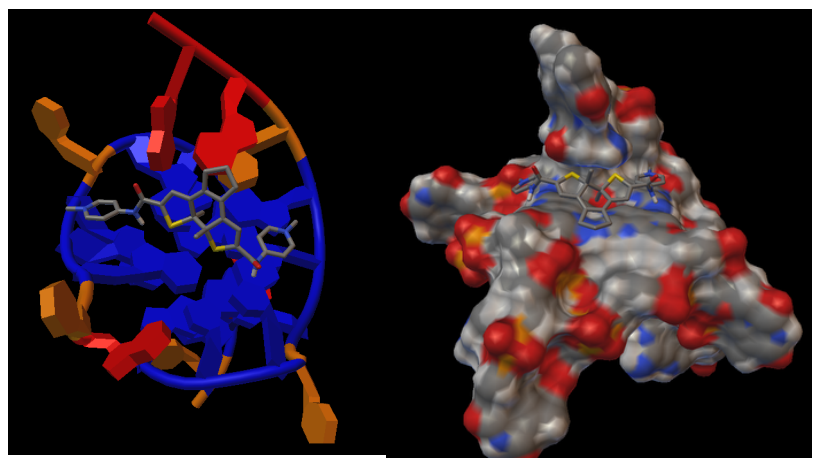	-7.3
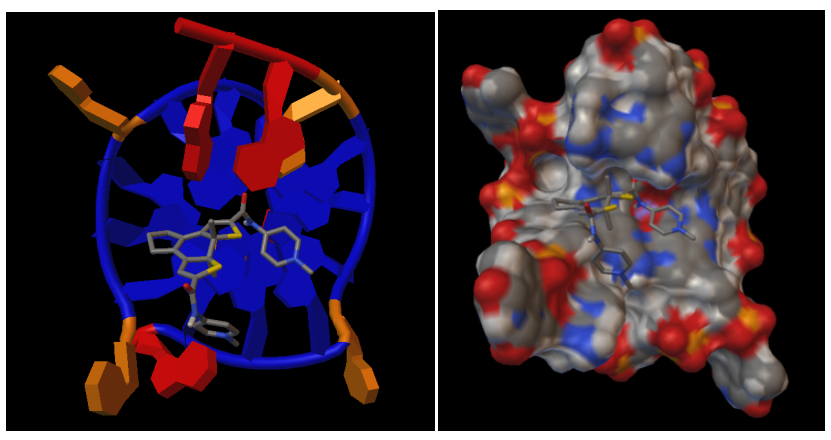	-7.4	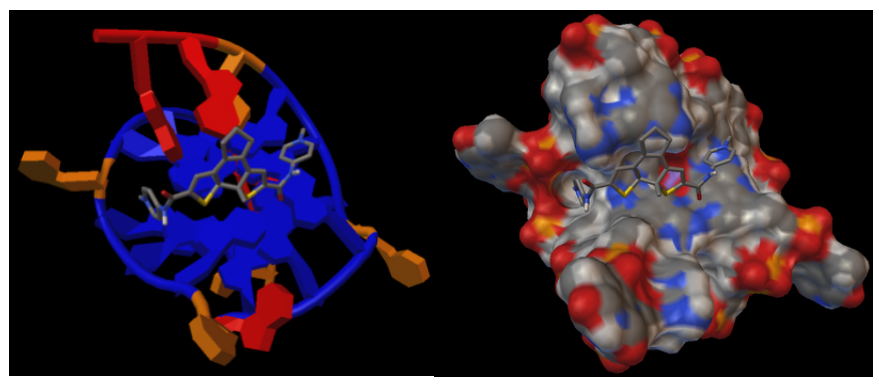	-7.3

Figure S24. The eight best-scoring (i.e. lowest-energy) poses for compound **2cl** when bound to the parallel c-Myc (PDB: 2L7V, 5'-TGAGGGTGGGTAGGGTGGGTAA-3') and corresponding binding energies. Each model is shown as a cartoon colored by base (A = red, T = orange, G = blue) with an overlaid molecular surface colored by atom type (C = grey, N = blue, O = red, S = orange). The **2cl** ligand is depicted using a stick representation.

Extended modelling discussion

For telo22, the eight best-scoring (i.e. lowest-energy) binding poses for both photo-isomers exhibited a range of potential groove-binding interactions (Figure 6 in main text, Figures S21 and S22). For the open isomer (**2o**), the most stable predicted binding modes were located in the rear or right-hand grooves, with the ligand's bent-shape and flexibility allowing for interactions with both groove and loop nucleotides (Figure S21). Few face-to-face π-stacking was observed when analysing **2o** best-scoring poses (e.g., with A7 in the top-right loop); instead, the ligand's binding was dominated by end-face and offset π-stacking interactions, primarily with loop bases A7 and A13, G8-10, G20-22 on the rear face, and G3-4 and G8-9 on the right face, alongside extensive contacts with the sugar-phosphate backbone within the groove and loop regions.

For the closed isomer (**2cl**), the most-stable binding modes were located in the right- or left-hand grooves, with the ligand's flatter and more rigid structure favoring interactions confined to a single groove, rather than bending toward the loop regions. No face-to-face π-stacking interactions was observed in the eight best-scoring models, with several end-face and offset π-stacking interactions identified (mainly with A1, T11 and T12 in the loop regions, G14-16, G20-21 in the left groove, and G2-G4 and G8-G10 in the right groove) as well as contacts with the sugar-phosphate backbone within the groove. The more peripheral interactions likely contribute to the lower affinity and reduced structural perturbations observed associated with the closed-form enriched **PSS** (Figure S22), while the greater flexibility of the open isomer allows for tighter and more substantial interactions with telo22 G4. The docking results suggest that the increased flexibility introduced by the extended amide linkage in **2** enables the formation of stable groove-binding interactions with the antiparallel structure. Additionally, the H-bonding capability of the amide may also contribute to the extensive sugar-phosphate backbone interactions observed, potentially explaining the higher affinity of **2o** compared to **1** observed experimentally (see Table1 in main text).

For c-Myc, both **2o** and **2cl** exhibited a variety of binding modes on either the top or bottom face of the G4 (Figure 6 in main text, Figures S22 and S23). It is worth noting that the structure used here for the docking (PDB: 2L7V)[6] contains one additional thymine in the c-Myc structure used for the biophysical experiments; however, this variation does not significantly alter the overall G4 fold.[3] For the **2o** isomer, the most stable binding modes were located on either the top or bottom face of the G4, with additional interactions with the loops or terminal nucleotides (Figure S22). As observed for telo22, the increased flexibility of the open isomer allowed it to form additional interactions within the G4 grooves. Interestingly, despite ligand binding to the open faces, no face-face π-stacking were observed in the best models, with interactions dominated by end-face and offset π-stacking (mainly with G7, G11 and G16 on top face, G9, G13, G18 and G22 on the bottom face, and G8 and G21 in the right groove) together with interactions with the sugar-phosphate backbone.

For the **2cl** isomer, the most-stable binding modes were also located on the top or bottom face of the G4, with the more rigid ligand structure failing to reach the grooves (Figure S22). Limited face-face π-stacking was observed (with e.g. G9 on the bottom face) between **2c** and the G4, with most interactions again resulting from end-face and offset π-stacking (mainly with G7, G11 and G16 on the top face and G9, G13, G18 and G22 on the bottom face) as well as from contacts with the sugar-phosphate backbone. Similarly to what observed for telo22, out docking results suggest that the enhanced flexibility of the open isomer allow for more effective and extensive interactions with c-Myc G4.

References.

1. P. Thordarson, *Chem Soc Rev*, 2011, **40**, 1305-1323.
2. Y. Wang and D. J. Patel, *Structure*, 1993, **1**, 263-282.
3. A. Ambrus, D. Chen, J. Dai, R. A. Jones and D. Yang, *Biochemistry*, 2005, **44**, 2048-2058.
4. GaussView, Version 6.1, Roy Dennington, Todd A. Keith, and John M. Millam, Semichem Inc., Shawnee Mission, KS, 2016
5. O. Trott and A. J. Olson, *J Comp Chem*, 2010, **31**, 455-461.
6. J. Dai, M. Carver, L. H. Hurley and D. Yang, *J Am Chem Soc*, 2011, **133**, 17673-17680.
